# Sex impacts treatment decisions in multiple sclerosis

**DOI:** 10.1007/s00415-024-12270-y

**Published:** 2024-03-05

**Authors:** Harald Hegen, Klaus Berek, Florian Deisenhammer, Thomas Berger, Christian Enzinger, Michael Guger, Jörg Kraus, Janette Walde, Franziska Di Pauli

**Affiliations:** 1 Department of Neurology, Medical University of Innsbruck, Anichstraße 35, 6020 Innsbruck, Austria; 2 Department of Neurology, Medical University of Vienna, Vienna, Austria; 3 Comprehensive Center for Clinical Neurosciences and Mental Health, Medical University of Vienna, Vienna, Austria; 4 Department of Neurology, Medical University of Graz, Graz, Austria; 5 Department of Neurology, Pyhrn-Eisenwurzen Hospital Steyr, Steyr, Austria; 6 Medical Faculty, Johannes Kepler University Linz, Linz, Austria; 7 Department of Laboratory Medicine, Paracelsus Medical University and Salzburger Landeskliniken, Salzburg, Austria; 8 Department of Neurology, Medical Faculty, Heinrich Heine University Düsseldorf, Düsseldorf, Germany; 9 Department of Statistics, Faculty of Economics and Statistics, University of Innsbruck, Innsbruck, Austria

**Keywords:** Multiple sclerosis, Gender, Sex, Treatment, Registry

## Abstract

**Background:**

Individual disease-modifying treatment (DMT) decisions might differ between female and male people with MS (pwMS).

**Objective:**

To identify sex-related differences in DMT strategies over the past decades in a real-world setting.

**Methods:**

In this cohort study, data from the Austrian Multiple Sclerosis Treatment Registry (AMSTR), a nationwide prospectively collected registry mandatory for reimbursement, were retrospectively analyzed. Of 4840 pwMS, those with relapsing–remitting MS, aged at least 18 years, who started DMT and had at least two clinical visits, were identified. At baseline, demographics, Expanded Disability Status Scale (EDSS) score, annualized relapse rate (ARR) in the prior 12 months and MRI lesion load were assessed. At follow-up, ARR, EDSS scores, and DMT were determined.

**Results:**

A total of 4224 pwMS were included into the study and had a median of 10 (IQR 5–18) clinical visits over an observation period of 3.5 (IQR 1.5–6.1) years. Multivariable Cox regression analysis revealed that the probability of DMT escalation due to relapse activity was lower in female than male pwMS (HR 4.1 vs. 8.3 per ARR). Probability of discontinuing moderate-effective DMT was higher in female pwMS when they were younger (HR 1.03 per year), and lower in male pwMS at higher age (HR 0.92). Similarly, female pwMS were more likely to stop highly effective DMT than male pwMS (HR 1.7). Among others, the most frequent reason for DMT discontinuation was family planning in female pwMS. All sex-related effects were independent of disease activity, such as MRI lesion load, baseline ARR or EDSS.

**Conclusions:**

Real-world treatment decisions are influenced by sex-related aspects. Awareness of these associations should prevent unwarranted differences in MS care.

**Supplementary Information:**

The online version contains supplementary material available at 10.1007/s00415-024-12270-y.

## Introduction

A female predominance in relapsing multiple sclerosis (RMS) is well known with a female: male ratio of 2.3–3.5:1 which even showed an increase in the last decades [1, 2]. To date, several differences in MS disease course depending on the biological sex were described, e.g., women are younger at disease onset [3], show a higher relapse activity [4], develop secondary progressive MS (SPMS) later during the disease course [5], and have slower disability progression [6]. Only a limited number of clinical trials performed sex-specific subgroup analysis regarding the efficacy of disease-modifying treatments (DMT), even though a recent meta-analysis found no clear sex-based difference of DMT response with regard to clinical outcomes [7].

Family planning, in particular pregnancy and lactation, around the use of DMT is an important issue in the care of people with MS (pwMS), especially in female pwMS, as the disease starts in young adulthood and, thus, at the age with childbearing potential [8, 9]. Maternal risk of disease worsening needs to be weighed against the fetal and new-born risk due to DMT exposure. While some DMTs are contraindicated during pregnancy and lactation, and re-activation of inflammatory MS disease activity might occur in case of DMT discontinuation, evidence increased in the last years for optimized strategies particular in women with high disease activity to reduce under-treatment and pregnancy-associated MS morbidity [9]. In addition, more safety data about fetal and new-born risk were gained.

Besides that, knowledge of patient management (reasonable or not) in real-world cohorts is of importance, as it creates awareness of daily clinical routine and may prevent unwarranted differences in MS care. Study results on sex-related differences in DMT prescriptions and treatment strategies in pwMS are scarce which is why we performed the present study.

## Methods

### Study design

In this cohort study, we retrospectively analyzed demographic, clinical, and para-clinical data from the Austrian Multiple Sclerosis Treatment registry (AMSTR). For details of the registry, we refer to Guger et al. [10]. Briefly, the AMSTR records data which have been prospectively entered into this database since 2006 during routine clinical visits from MS centers throughout Austria. This includes, at baseline, date of clinical onset of MS, number of relapses in the prior 12 months, Expanded Disability Status Scale (EDSS) score, load of hyperintense lesions on T2-weighted MRI (> 9, ≤ 9), and the use of previous DMT. At follow-up visits, occurrence of relapses, EDSS score, DMT, and adverse events (AE) are required to be documented every 3–6 months. In case of DMT change, reasons are provided (e.g., family planning or MRI activity). The dataset contains records from 89 centers, dated from August 3, 2006 to November 10, 2020.

### Patients’ inclusion criteria

Patients were eligible for inclusion if they had a diagnosis of relapsing–remitting MS [11–13], were aged at least 18 years at start of DMT, with a minimum of 2 consecutive visits. Eligible pwMS were categorized as starting high-efficacy DMT (hDMT), i.e. ocrelizumab (OCR), alemtuzumab (ALZ), natalizumab (NTZ), fingolimod (FTY), cladribine (CLB), or moderate-efficacy DMT (mDMT), i.e. dimethyl fumarate or teriflunomide [14–17]. All DMTs were given according to their label.

### Definition of disease activity

A relapse was defined as patient-reported symptoms and objectively confirmed neurological signs typical for an acute central nervous system inflammatory demyelinating event with duration of at least 24 h in the absence of fever or infection and separated from the last relapse by at least 30 days [11]. Annualized relapse rate (ARR) was calculated as the sum of relapses per observation period in years.

Disability (EDSS) progression was defined as an increase of EDSS score from baseline of at least 1.5 points if baseline EDSS was 0, 1.0 point if baseline EDSS was ≥ 1.0 and ≤ 5.0, and 0.5 points if baseline EDSS was ≥ 5.5 [18].

MRI activity was defined as new or enlarging T2 lesions [19].

### Definition of treatment strategies

DMT escalation was defined as the patients’ first switch from mDMT to hDMT. DMT de-escalation was the patients’ first switch from hDMT to mDMT. DMT discontinuation included patients who stopped either mDMT or hDMT.

### Objective

We aimed to identify differences in treatment between female and male pwMS including differences in treatment escalation, de-escalation, and discontinuation.

### Primary outcomes

The primary outcomes were time to DMT escalation (‘Question 1’), time to discontinuation of mDMT (‘Question 2’), time to DMT de-escalation (‘Question 3’), and time to discontinuation of hDMT (‘Question 4’). These time periods were calculated as the difference between baseline (start of first registry treatment) and treatment change.

### Secondary outcomes

Secondary outcomes subsumed i) time to DMT initiation and ii) initiation of mDMT versus hDMT.

### Statistical analysis

Continuous variables were displayed as median and interquartile range (IQR). Categorical variables were shown as frequencies and percentages.

We used statistical analyses to identify the impact of sex on treatment strategies adjusting for age (years), disease duration (years), DMT (categorical variable), prior ARR, baseline EDSS, baseline MRI T2L load (> 9, ≤ 9), pre-treatment (yes, no), EDSS progression (yes, no), and ARR on DMT. For the binary variable, mDMT vs. hDMT, logistic regression was used. For time to treatment initiation (time between disease onset and start of registry treatment), a generalized linear model (GLM) with gamma distribution and log link was used taking into account the skewness of the dependent variable distribution. Cox regression was used for time to escalation (Question 1), time to de-escalation (Question 3), and time to treatment discontinuation (Question 2 and 4). For the Cox regression with the dependent variable time to escalation, we used MRI activity during DMT as additional co-variable.

Our focus was on the sex effect, so interaction effects of sex with the independent variables were selected for all models in addition to the main effects via Akaike information criterion (AIC) [20]. If the interaction effects were not improving the AIC, they were not included in the final model.

We checked all models for multicollinearity with the variance inflation factor[21]. Coefficients ($\beta$) and 95% confidential intervals (CI) were presented as the main output of these models. As quality measure for the GLMs the Cox–Snell pseudo *R*^2^ measure is provided, for the Cox regressions the R-squared measure based on the partial likelihood ratio statistic [22]. Additionally, we performed sensitivity analyses. We ran regression analyses (for the primary outcomes) depending on DMT start, where we split our cohort into a part covering the last 5 years vs. before.

A posteriori power analyses for the above-mentioned multivariable models with binary and non-binary covariates were computed. We fixed the type one error rate at 5% and used effect sizes as revealed by the models. Either we used the actual sample size to calculate the a posteriori power, or set the power to 0.8 to compute the necessary sample size.

A *p* value < 0.05 was considered statistically significant. Bonferroni–Holm correction for multiple testing was performed [23]. All analyses were done using the statistical software R [24] with the “powerSurvEpi” package [25].

### Ethics

The AMSTR is approved by the ethical committee of the Medical University of Vienna (Approval number 2096/2013) and the Medical University of Innsbruck (Approval number 1235/2020).

## Results

**Fig. 1:**
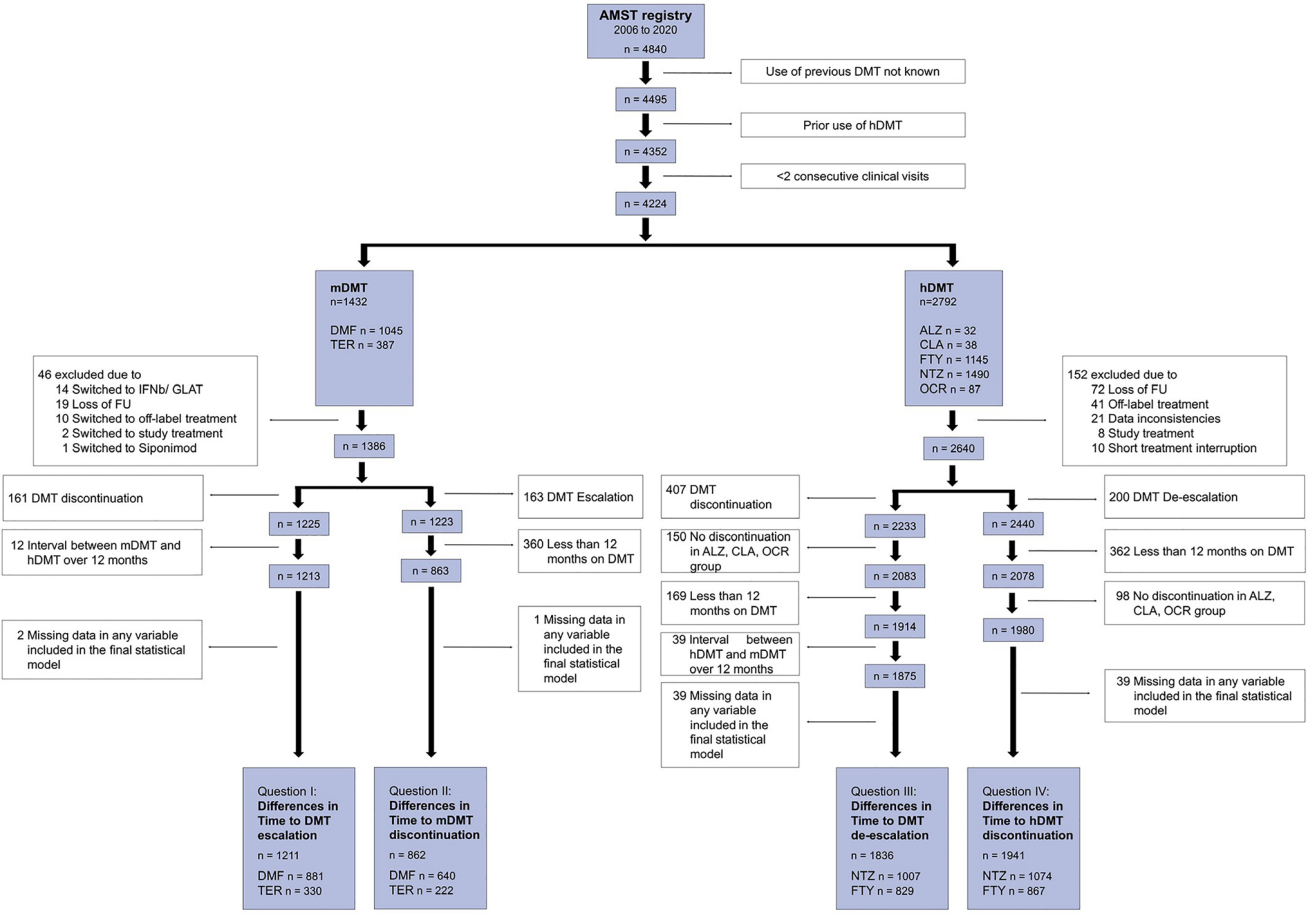
Patient selected by various inclusion criteria. *ALZ*, Alemtuzumab; *AMST*, Austrian Multiple Sclerosis Treatment; *CLA*, cladribine; *DMF*, dimethyl fumarate; *DMT*, disease-modifying treatment; *FTY*, fingolimod; *FU*, follow-up; *GLAT*, glatiramer acetate; *hDMT*, high-efficacy DMT; *mDMT*, moderate-efficacy DMT; *IFN*, interferon-beta; *NTZ*, natalizumab; *OCR*, ocrelizumab; *TER*, teriflunomide

**Fig. 2:**
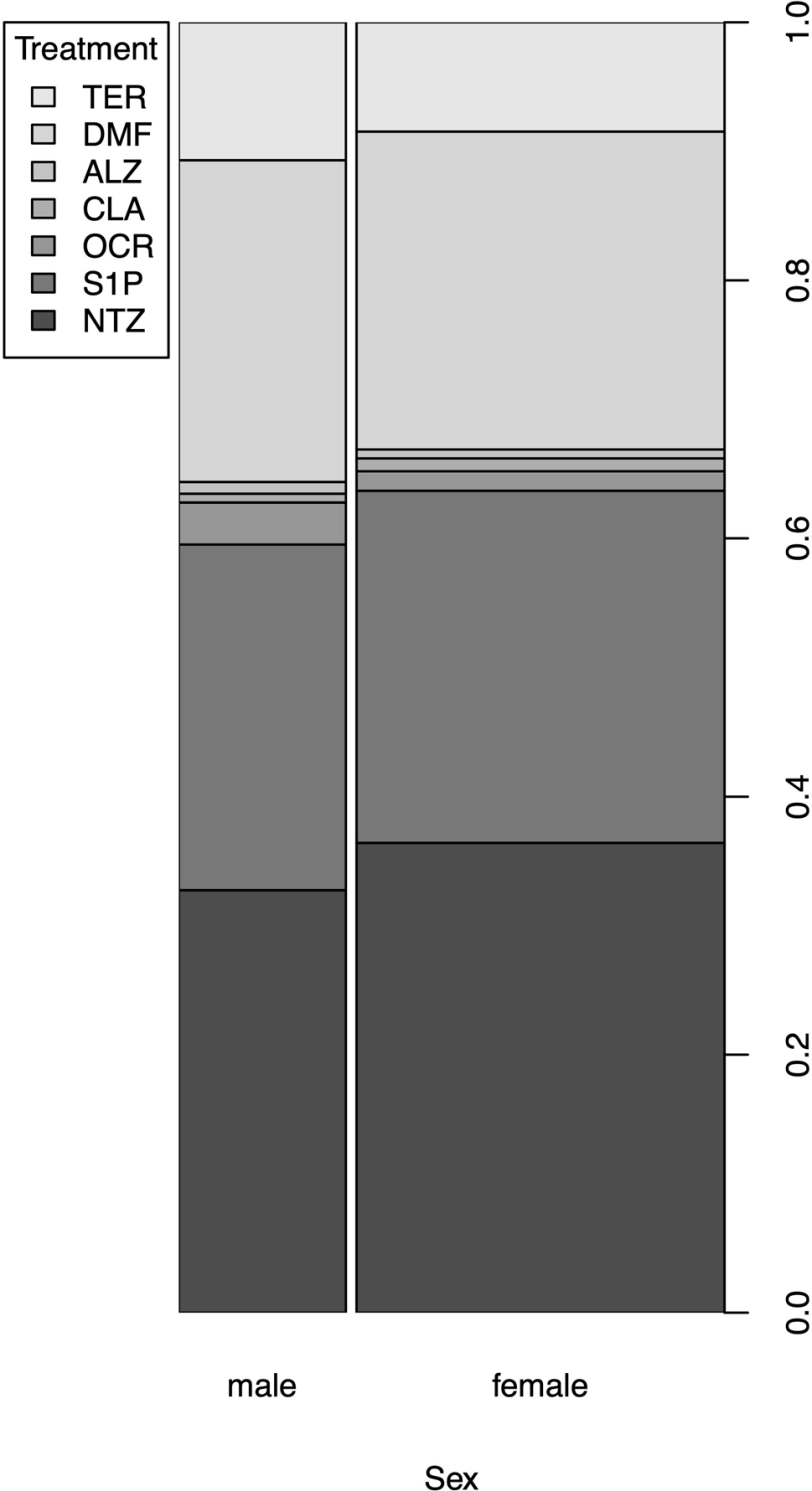
Different DMT in women and men at baseline. *ALZ*, Alemtuzumab; *CLA*, cladribine; *DMF*, dimethyl fumarate; *DMT*, disease-modifying treatment; *NTZ*, natalizumab; *OCR*, ocrelizumab; *S1P*, sphingosine-1-phosphate receptor modulator; *TER*, teriflunomide

Of 4840 pwMS available in the AMSTR, a total of 4224 (87%) were included in this study and had at median of 10 (IQR 5–18) visits over an observation period of 3.5 (IQR 1.5–6.1) years. At baseline, 2792 pwMS received hDMT, while 1432 pwMS received mDMT (Fig. 1). Frequency of mDMT and hDMT were similarly distributed between women and men (Table e-1, Table e-2, Fig. 2). Time to start of DMT was associated with various disease activity measures. Female pwMS had a longer time to DMT start independent of age, clinical disease activity (including ARR before and EDSS score at treatment start), MRI lesion load, and the prior administration of DMT (Table e-3, Fig. e-1).

### Relapses in women are a weaker trigger for DMT escalation.

Of 1211 pwMS receiving mDMT (Fig. 1), 149 (12.3%) were escalated to hDMT (Table e-4) due to various reasons, 88 (59%) had relapses, 16 (11%) isolated MRI activity and 6 (4%) patients had isolated EDSS progression. Twenty-nine (19%) patients reported AEs and 10 (7%) patients other causes for DMT escalation. Distribution of demographic and clinical characteristics between female and male pwMS is given in Table e-5.

**Fig. 3:**
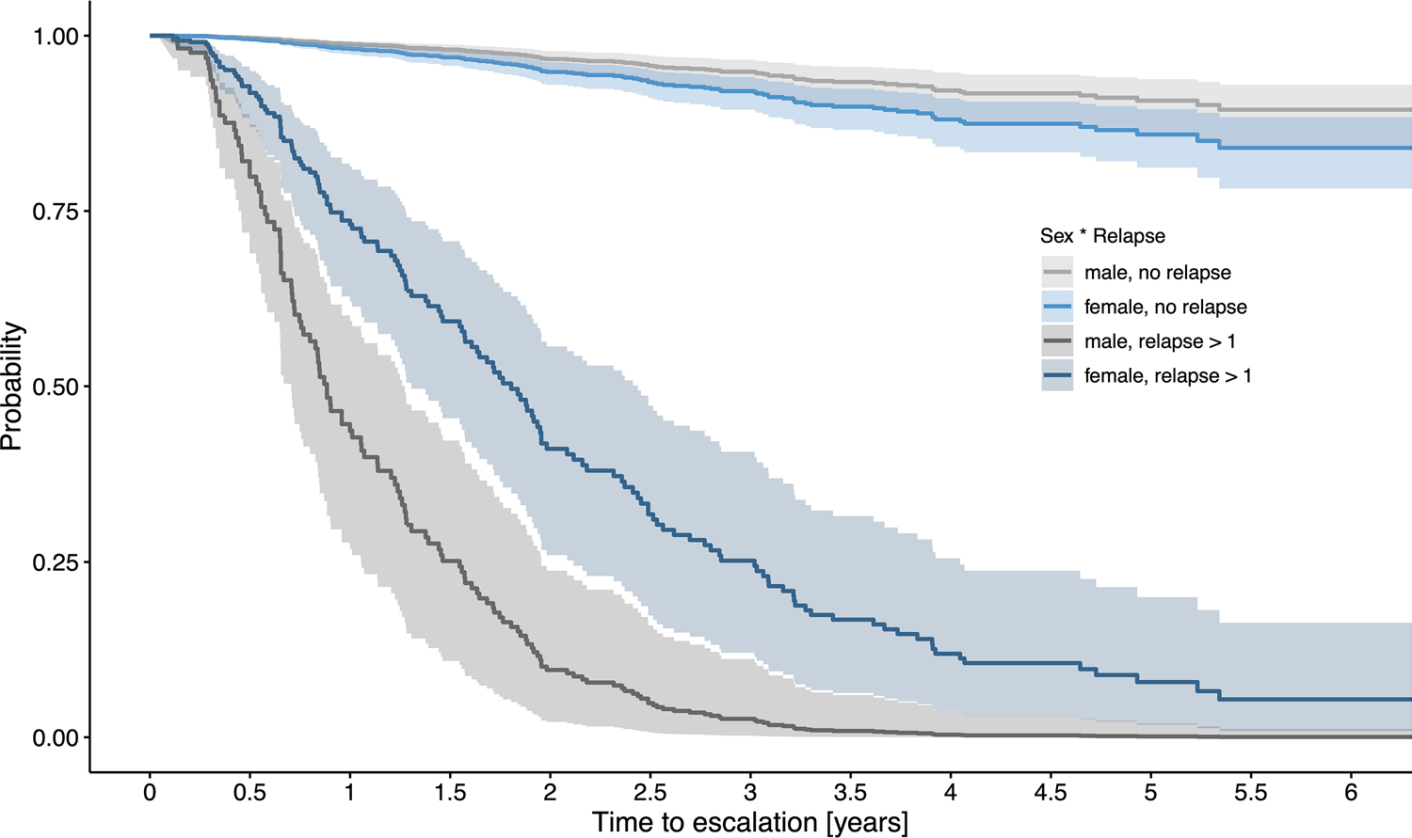
Time to DMT escalation depending on patients’ sex. To visualize the interaction effect of sex and ARR on DMT on the probability of DMT escalation, we computed the estimated Cox regression survival probabilities separately for male and female pwMS according to the occurrence of relapse (no relapse versus relapse > 1). In addition, DMT was set to “DMF”, pre-treatment “yes”, baseline MRI T2 lesion load “ > 9” and EDSS progression “no”. All other parameters (age, disease duration, pre-ARR, baseline EDSS) were set to their median values. *ARR*, annualized relapse rate; *DMT*, disease-modifying treatment; *EDSS*, Expanded Disability Status Scale; DMF, dimethyl fumarate

Multivariable Cox regression analysis showed that various disease activity-related variables, such as ARR during treatment (*β* = 2.1, 95% CI [1.8, 2.5]), EDSS progression (*β* = *0*.4, [0.1, 0.8]) and occurrence of MRI activity (*β* = 3.2, [2.5, 3.8]), were predictors of DMT escalation. Besides that, also a sex-related effect on the time to DMT escalation was observed. While an increase in ARR by 1 means an increased hazard ratio (HR) of approximately 8 to escalate DMT in men (*β* = 2.1, [1.8, 2.5]), the increased HR was only approximately 4 in women (*β* = 1.4, [1.2, 1.6]) (Table 1, Fig. 3).

**Table 1:** Cox regression analysis identifying predictors of DMT escalation (Question 1)

	Coefficient	SE	P value	95% CI		Hazard ratio
Sex (female, ref: male)	0.446	0.232	0.055	− 0.010	0.902	1.562
Age [years]	− 0.021	0.010	0.036	− 0.041	− 0.001	0.979
Disease duration [years]	− 0.025	0.015	0.099	− 0.056	0.005	0.975
Pre-ARR^†^	0.004	0.107	0.973	− 0.206	0.214	1.004
EDSS^§^	0.144	0.072	0.046	0.003	0.286	1.155
MRI T2LL^§^ (> 9, ref: ≤ 9)	− 0.021	0.235	0.930	− 0.482	0.441	0.979
Pre-treatment^¶^ (yes, ref: no)	0.017	0.180	0.926	− 0.337	0.370	1.017
DMT (DMF, ref: TER)	− 0.043	0.191	0.824	− 0.417	0.332	0.958
ARR on DMT	2.117	0.172	** < 0.001**	1.779	2.455	8.309
EDSS progression (yes, ref: no)	0.417	0.177	0.019	0.069	0.765	1.517
MRI activity during DMT (yes, ref: no)	3.166	0.316	** < 0.001**	2.546	3.786	23.709
Sex : ARR on DMT	− 0.708	0.186	** < 0.001**	− 1.073	− 0.344	0.493

Bold *p* values hold with Bonferroni–Holm correction

*ARR*, annualized relapse rate; *CI*, confidence interval; *DMF*, dimethyl fumarate; *DMT*, disease-modifying treatment; *EDSS*, Expanded Disability Status Scale; *MRI*, magnetic resonance imaging; *ref*, reference; *SE*, standard error; *T2LL*, T2 lesions load; *TER*, teriflunomide

*R*-squared: 0.836

^§^These variables were assessed at baseline

^†^ARR was determined in the 12 months prior to baseline

^¶^ Pre-treatment included interferon-beta and/ or glatiramer acetate

[] shows units and () indicates reference categories

“:” denotes interaction effects between variables

### Moderate-efficacy DMT were more likely discontinued in women at younger age.

Of 862 pwMS who were on mDMT for at least 12 months (Fig. 1), 73 (8.5%) stopped DMT (Table e-6). Distribution of demographic and clinical characteristics between female and male pwMS is given in Table e-7.

**Fig. 4:**
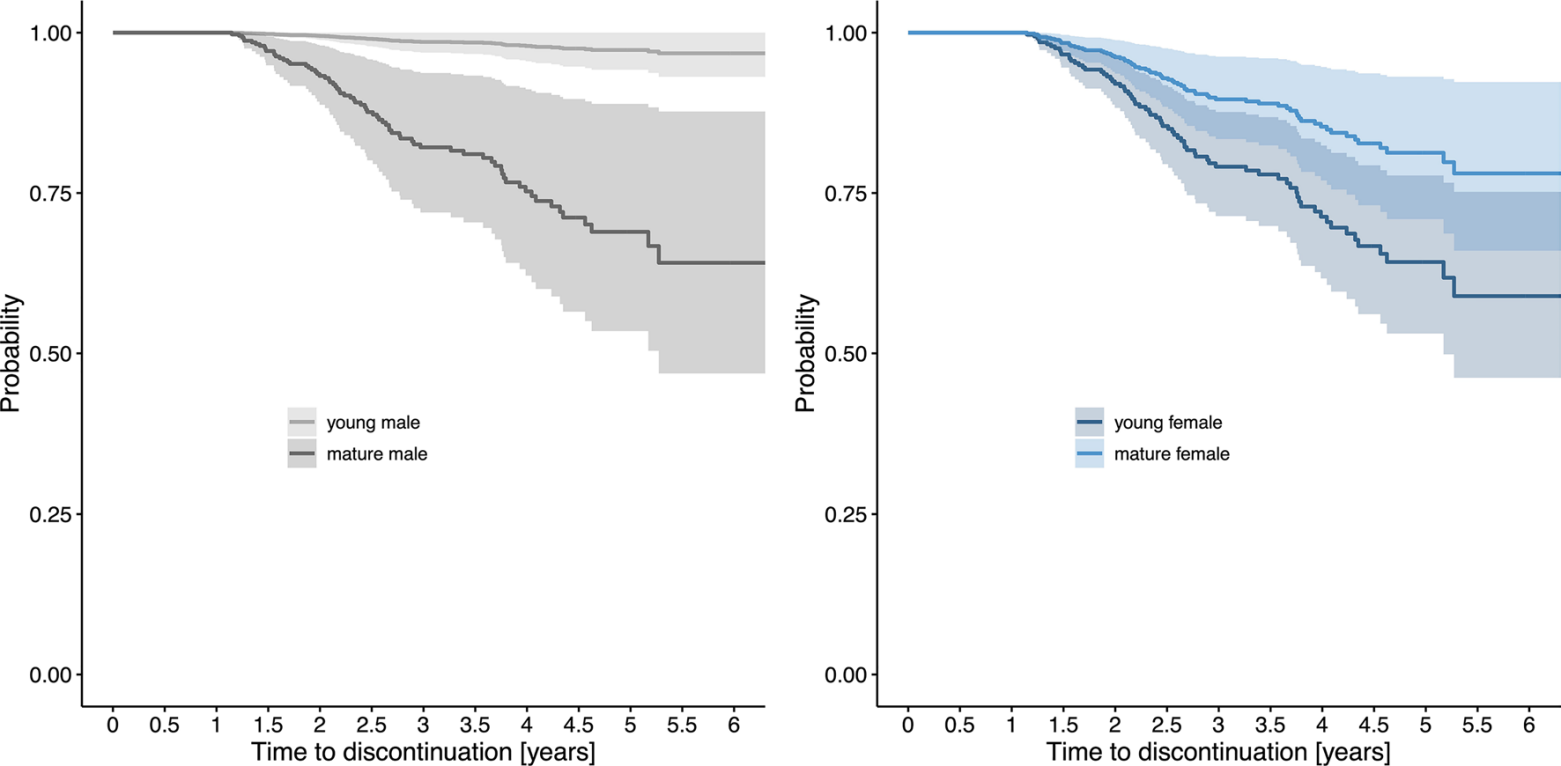
Time to mDMT discontinuation depending on patients’ sex and age. To visualize the interaction effect of sex and age on the probability of treatment discontinuation, we computed the estimated Cox regression survival probabilities for male (left panel) and female pwMS (right panel), each separately for mature (age set at 55 years) and young pwMS (age set at 25 years). In addition, DMT was set to “DMF”, pre-treatment “yes”, baseline MRI T2 lesion load “ > 9”, EDSS progression “no” and ARR on DMT “0”. All other parameters (disease duration, pre-ARR, baseline EDSS) were set to their median values. *ARR*, annualized relapse rate; *DMF*, dimethyl fumarate; *DMT*, disease-modifying treatment; *EDSS*, Expanded Disability Status Scale; *mDMT*, moderate-efficacy DMT

Multivariable Cox regression analysis showed that lower ARR (*β* = − 1.7, [− 3.3, − 0.1]) as well as EDSS progression (*β* = *0*.8, [0.1–1.4,]) was associated with treatment termination. Female pwMS had a higher HR of approximately 3 for stopping treatment (*β* = 1.2, [0.4, 2.0]). Additionally, there was a different impact of age on the probability for treatment discontinuation depending on sex. The younger female pwMS were, the higher the HR for treatment discontinuation (*β* = − 0.03, [− 0.06, 0.0]). Contrary, male pwMS had overall a lower HR for stopping treatment, but the HR increased with increasing age (*β* = 0.09, [0.03, 0.14]) (Table 2, Fig. 4).

**Table 2:** Cox regression analysis identifying predictors of early moderate-efficacy DMT discontinuation (*Question 2*)

	Coefficient	SE	*P* value	95% CI		Hazard ratio
Sex (female, ref: male)	1.214	0.405	**0.003**	0.421	2.007	3.366
Age^^^ [years]	0.087	0.028	**0.002**	0.032	0.142	1.091
Disease duration [years]	− 0.031	0.020	0.131	− 0.071	0.009	0.970
Pre-ARR^†^	− 0.027	0.159	0.864	− 0.338	0.284	0.973
EDSS^§^	0.132	0.097	0.172	− 0.058	0.322	1.141
MRI T2LL^§^ (> 9, ref: ≤ 9)	− 0.032	0.329	0.922	− 0.678	0.613	0.968
Pre-treatment^¶^ (yes, ref: no)	0.337	0.252	0.181	− 0.157	0.830	1.400
DMT (DMF, ref: TER)	0.617	0.316	0.051	− 0.003	1.237	1.853
ARR on DMT	− 1.674	0.823	0.042	− 3.287	− 0.060	0.188
EDSS progression (yes, ref: no)	0.773	0.328	** < 0.001**	0.131	1.416	2.167
Sex : Age	− 0.112	0.029	** < 0.001**	− 0.170	− 0.055	0.894

*R*-squared based on the partial likelihood ratio statistic under the Cox model: 0.453

Bold p-values hold with Bonferroni–Holm correction

*ARR*, annualized relapse rate; *CI*, confidence interval; *DMF*, dimethyl fumarate; *DMT*, disease-modifying treatment; *EDSS*, Expanded Disability Status Scale; *MRI*, magnetic resonance imaging; *ref*, reference; *SE*, standard error; *T2LL*, T2 lesions load; *TER*, teriflunomide

^§^These variables were assessed at baseline

^†^ARR was determined in the 12 months prior to baseline

^¶^Pre-treatment included interferon-beta and/ or glatiramer acetate

^Due to multicollinearity with the interaction effect age was demeaned

[] shows units and () indicates reference categories

“:” denotes interaction effects between variables

Reasons for stopping mDMT were family planning (*n* = 24; 33%), patient`s request (*n* = 19; 26%) disease stability (*n* = 8; 11%), and disease progression (*n* = 5; 7%). In 17 pwMS (26%), DMT was stopped due to AEs. Compared to the average female predominance of approximately 70%, family planning as reason for mDMT stopping was dominated by female pwMS (100%), while disease progression by male sex (80%) (Table e-8).

### DMT de-escalation at similar frequencies in women and men with MS

Of 1836 pwMS receiving NTZ or FTY over a period of at least 12 months, 78 (4.2%) were deescalated to a mDMT. Patients receiving ALZ, CLB, or OCR were not included in the analysis due to lack of de-escalating patients (Fig. 1). Twenty-nine (37%) switched from FTY and 49 (63%) from NTZ (Table e-9). Distribution of demographic and clinical characteristics between female and male pwMS is given in Table e-10.

Most frequent reasons for de-escalation were JCV positivity (*n* = 38; 49%) and adverse events (*n* = 27; 36%) followed by pwMS’ request (*n* = 8; 11%), transition to SPMS (*n* = 4; 4%), and other reasons (*n* = 1; 1%).

Multivariable Cox regression analysis showed that pwMS with lack of EDSS progression (*β* = − 0.7, [− 1.2, − 0.2]) was more likely de-escalated (Table 3). No interaction effects with sex were statistically significant.

**Table 3:** Cox regression analysis identifying predictors of DMT de-escalation (*Question 3*)

	Coefficient	SE	P value	95%-CI		Hazard ratio
Sex (female, ref: male)	− 0.385	0.235	0.101	− 0.845	0.075	0.680
Age [years]	0.014	0.014	0.326	− 0.014	0.042	1.014
Disease duration [years]	0.008	0.020	0.709	− 0.032	0.047	1.008
Pre-ARR^†^	− 0.060	0.102	0.558	− 0.260	0.140	0.942
EDSS^§^	− 0.069	0.090	0.443	− 0.246	0.107	0.933
MRI T2LL^§^ (> 9, ref: ≤ 9)	0.076	0.466	0.871	− 0.838	0.990	1.079
Pre-treatment^¶^ (yes, ref: no)	0.119	0.379	0.754	− 0.624	0.862	1.126
DMT (NTZ, ref: FTY)	− 0.058	0.273	0.831	− 0.593	0.477	0.943
ARR on DMT	0.292	0.437	0.504	− 0.565	1.148	1.339
EDSS progression (yes, ref: no)	− 0.719	0.266	0.007	− 1.240	− 0.197	0.487

*R* square: 0.160

Bold *p* values hold with Bonferroni–Holm correction

*ARR*, annualized relapse rate; *CI*, confidence interval; *DMT*, disease-modifying treatment; *EDSS*, Expanded Disability Status Scale; *FTY*, fingolimod; *MRI*, magnetic resonance imaging; *NTZ*, natalizumab; *ref*, reference; *SE*, standard error; *T2LL*, T2 lesions load

^§^These variables were assessed at baseline

^†^ARR was determined in the 12 months prior to baseline

^¶^Pre-treatment included interferon-beta and/or glatiramer acetate

[] shows units and () indicates reference categories

### Women with MS more frequently stopped high-efficacy DMT

Of 1941 pwMS with hDMT (Fig. 1), 231 (12%) stopped DMT (Table e-11). Distribution of demographic and clinical characteristics between female and male pwMS is given in Table e-12.

Multivariable Cox regression revealed that EDSS progression (*β* = 0.6, [0.3, 0.9]) during DMT was associated with DMT stop. Higher relapse rate was associated with lower HR for DMT stop (*β* = − 1.0, [− 1.7, − 0.2]); however, this effect was reversed in a subgroup of pwMS who requested DMT stop due to various reasons including adverse events, family planning or specific patient request (Table 4). Overall, females had an increased HR of 1.7 to quit hDMT (*β* = 0.5, [0.2, 0.9]).

**Table 4:** Cox regression analysis identifying predictors of early hDMT discontinuation (*Question 4*)

	Coefficient	SE	*P* value	95% CI		Hazard ratio
Sex (female, ref: male)	0.542	0.169	**0.001**	0.211	0.873	1.719
Age [years]	0.001	0.008	0.927	− 0.015	0.017	1.001
Disease duration [years]	− 0.021	0.012	0.090	− 0.045	0.003	0.979
Pre-ARR^†^	− 0.034	0.061	0.569	− 0.153	0.084	0.966
EDSS^§^	0.163	0.047	** < 0.001**	0.070	0.255	1.176
MRI T2LL^§^ (> 9, ref: ≤ 9)	0.030	0.280	0.914	− 0.518	0.579	1.031
Pre-treatment^¶^ (yes, ref: no)	− 0.236	0.190	0.217	− 0.608	0.138	0.791
DMT (NTZ, ref: FTY)	− 0.009	0.160	0.956	− 0.322	0.304	0.991
ARR on DMT^#^	− 0.957	0.377	0.011	− 1.697	− 0.217	0.384
EDSS progression (yes, ref: no)	0.634	0.152	** < 0.001**	0.337	0.932	1.886

*R* squared: 0.596

Bold *p* values hold with Bonferroni–Holm correction

*ARR*, annualized relapse rate; *DMT*, disease-modifying treatment; *CI*, confidence interval; *EDSS*, Expanded Disability Status Scale; *FTY*, fingolimod; *MRI*, magnetic resonance imaging; *NTZ*, natalizumab; *ref*, reference; *SE*, standard error; *T2LL*, T2 lesions load

^§^These variables were assessed at baseline

^†^ARR was determined in the 12 months prior to baseline

^¶^Pre-treatment included interferon-beta and/ or glatiramer acetate

^#^Additionally, interaction between the variable “ARR on DMT” and different “reasons for DMT stop” (family planning, adverse events, patient request) had to be considered due to confounding

[] shows units and () indicates reference categories

The most frequent reasons for stopping hDMT were JCV positivity (*n* = 32; 14%), adverse events (*n* = 31; 13%), progression (*n* = 31; 13%), pwMS’ request (*n* = 43; 19%), family planning (*n* = 74; 32%), neutralizing antibodies (*n* = 1; 0.4%), progressive multifocal leukoencephalopathy (*n* = 7; 3%), stability (*n* = 3; 1%), and other reasons (*n* = 9; 4%). Compared to the average female predominance of approximately 70%, family planning was the reason for hDMT stopping clearly dominated by a sex effect, as all of these individuals were female (Table e-13).

## Discussion

Real-world data regarding sex-related differences in MS treatment strategies are scarce. The results of our study should create awareness of potential sex-related treatment differences and avoid unwarranted differences in MS care.

In our study, we observed that female pwMS had a longer time to DMT start independent of age, clinical disease activity (ARR, EDSS) and MRI lesion load. Female pwMS also had delayed treatment escalation despite relapses compared to male pwMS. An increase in ARR by 1 means an approximately 8 times higher probability to escalate DMT in men, but only 4 times higher probability in women. In addition, discontinuation of mDMT was more likely in females, especially when they were younger, and the probability to stop hDMT was higher in female compared to male pwMS.

Similar results were found in the Danish Multiple Sclerosis Registry [26]. Men were more likely to receive hDMT right from the start (odds ratio 1.53) and were more likely to be escalated to hDMT (odds ratio 2.03) than women. Sex differences in treatment discontinuation or de-escalation were not examined in this study. Supporting the results of our study, further work reported that women received hDMT less frequently than men (odds ratio 0.92) [27]. In addition, consistent with our results, this study showed that younger age, higher relapse rate, and higher EDSS scores were also associated with a higher likelihood of hDMT [27]. Furthermore, in 499 pwMS receiving interferon-beta as first DMT, interferon-beta discontinuation was more frequent in female pwMS than in male pwMS (HR 1.42) [28].These results were confirmed by another study, which showed that women were more likely to stop interferon-beta or glatiramer acetate initiated after MS diagnosis than men [29]. The limitation of these studies is that there are no data for hDMT discontinuation.

One reason for different treatment strategies and different weighting of disease activity is family planning. In our study, family planning was one of the most common reasons to discontinue a DMT. The AMSTR started in 2006 [10]. At that time and in subsequent years, there was insufficient evidence of fetal risk and use of DMT. Thus, all DMT had a contraindication in pregnancy and were discontinued before conception or in early pregnancy [30]. Due to increasing data from pregnancy registries, it is now possible to continue certain therapies depending on the activity of the disease and individual benefit–risk evaluation [9].

E.g., in the case of NTZ treatment, bearing a high risk of disease reactivation or even rebound activity, treatment interruption should be avoided to prevent women from further, potentially debilitating disease activity [31]. Whereas in pwMS treated with NTZ (depending on the activity of the disease), it is possible to continue the therapy with a different treatment interval, S1P receptor modulators (where rebound or a high risk of disease reactivation is also described [32]) must be discontinued due to their potential teratogenic effect [33]. Few data are available regarding treatment with CD20 antibodies and pregnancy. Although continuous administration of CD20 antibodies is necessary to suppress disease activity, no excessive disease activity after discontinuation has been observed so far [34]. In contrast to hDMT, discontinuation of mDMT in women with a stable course of disease without clinical or radiological disease activity is considered relatively safe [35].

Until now, a different effectiveness of the different DMT depending on sex could not be proven and does not justify different treatment [2, 36]. However, in clinical studies, sex subgroup analyses are still rarely done and a definitive statement regarding a different treatment response in female and male pwMS is not possible yet with certainty [7, 37]. In clinical trials, strict eligibility criteria such as consent to contraception during the study period may not reflect the diverse patient population encountered in routine clinical practice. In addition to clinical trials, an increasing number of real-world studies are available, but these mainly focus on different treatment strategies such as the early use of highly effective therapies versus their late use or the relapse rate after stopping DMT. Since the propensity score method is often used in these studies to reduce sex-selection bias through matching, they do not allow any statement in regard of sex-related differences in treatment strategies. On the other hand, the real-world register data shown here enables a representative overview of a sex-bias in the treatment of MS. Especially since an entry in the registry is required for reimbursement and a special quality-related feature of the AMSTR is that data where external and independent monitored. This guarantees improved acquisition and completeness of the data, and the representativeness of the cohort.

There are some limitations to this study. The first limitation of the study is that the AMSTR has existed since the first hDMT was approved and therefore previously approved therapies (interferon-beta preparations, glatiramer acetate) are not captured in this registry. This also explains the high percentage (66%) of hDMT in the AMSTR, as many patients were pre-treated with interferon-beta or glatiramer acetate. Also, the data on the discontinuation of newer treatments such as CD20 antibodies or the de-escalation of these are therefore very limited. Furthermore, in case of treatment discontinuation, no further follow-up data are available, unless patients re-start any treatment again in future. Although we included a set of relevant co-variables in the multivariable analyses, other potential confounders might not have been covered, e.g. MRI data were only available in patients with DMT escalation, and not available in patients before DMT discontinuation or de-escalation. Such information might impact on treatment decision making. Also, after identifying a sex-related effect, e.g., on DMT stopping, we could only describe the possible causes, such as family planning as a main reason in approximately 30% of DMT stoppers and that all of them were females. This observation provides an exploratory hypothesis for the observed sex-effect, but could not be included in the multivariable analysis, as this information was not available for pwMS continuing DMT. However, subgroup analyses confirmed this hypothesis, as after exclusion of female pwMS with family planning, the sex-related effect was lost (Table e-14 and Table e-15). Furthermore, the reason “family planning” was not available for all research questions (e.g., time to DMT start), and a differentiation between wish for pregnancy, pregnancy and lactation was not possible due to lack of data.

We also performed sensitivity analyses (Table e-16, e-17, e-18 and e-19). We did not observe a cohort effect, when we compared pwMS starting DMT before or after 2015, i.e., statistically significant effects of co-variables remained qualitatively the same (for the primary outcomes). There was only one exception. After 2015, the frequency of pwMS discontinuing hDMT was lower (14% vs. 7%). This might be attributed to different treatment strategies in the last years due to the risk of rebound phenomenon. Whether the observed sex-related effect (main effect) remains after 2015, evidence is not unambiguous yet. A larger time series would be necessary.

Besides the sex-related effects as discussed above, there might be additional sex-related effects that were not uncovered in our study due to low statistical power, e.g., the sex effect on time to DMT de-escalation (showing a clinically relevant coefficient of − 0.4 and a posteriori power of 0.5). There is one exception. The lacking effect of sex on time to DMT escalation is indeed supported by the power analysis. A posteriori power analyses for the multivariable models are given in Table e-20, e-21, e-22 and e-23.

Since several studies have shown an increasing risk of disability due to relapses during pregnancy, awareness of the conscious or partly unconscious different treatment of women and men is of great importance for the treating neurologist. Our study results should increase the awareness of sex-related treatment differences, thus, prevent unwarranted treatment decisions and eventually prevent further disease activity and morbidity especially in women. Despite the clear results of our study, a replication in a different cohort would be desirable, where also some of the above-mentioned limitations could be addressed, e.g., where patients on immunodepleting agents such as CD20 antibodies are included.

### Supplementary Information

Below is the link to the electronic supplementary material.

Supplementary file1 (PDF 2231 kb)

## Abbreviations

AE: Adverse events
ALZ: Alemtuzumab
AMSTR: Austrian Multiple Sclerosis Treatment Registry
ARR: Annualized relapse rate
BL: Baseline
CLB: Cladribine
DMF: Dimethyl fumarate
DMT: Disease-modifying treatment
EDSS: Expanded Disability Status Scale
FTY: Fingolimod
FU: Follow-up
GLAT: Glatiramer acetate
GLM: Generalized linear model
hDMT: High-efficacy DMT
IFN: Interferon-beta
mDMT: Moderate-efficacy DMT
MRI: Magnetic resonance imaging
NTZ: Natalizumab
OCR: Ocrelizumab
pwMS: People with multiple sclerosis
RMS: Relapsing multiple sclerosis
T2LL: T2 lesions load
TER: Teriflunomide

## References

[1] Harbo HF, Gold R, Tintoré M (2013). Sex and gender issues in multiple sclerosis. Ther Adv Neurol Disord.

[2] Gilli F, DiSano KD, Pachner AR (2020). SeXX matters in multiple sclerosis. Front Neurol.

[3] Cossburn M, Ingram G, Hirst C, Ben-Shlomo Y, Pickersgill TP, Robertson NP (2012). Age at onset as a determinant of presenting phenotype and initial relapse recovery in multiple sclerosis. Mult Scler.

[4] Kalincik T, Vivek V, Jokubaitis V, Lechner-Scott J, Trojano M, Izquierdo G, Lugaresi A, Grand'maison F, Hupperts R, Oreja-Guevara C, Bergamaschi R, Iuliano G, Alroughani R, Van Pesch V, Amato MP, Slee M, Verheul F, Fernandez-Bolanos R, Fiol M, Spitaleri DL, Cristiano E, Gray O, Cabrera-Gomez JA, Shaygannejad V, Herbert J, Vucic S, Needham M, Petkovska-Boskova T, Sirbu CA, Duquette P, Girard M, Grammond P, Boz C, Giuliani G, Rio ME, Barnett M, Flechter S, Moore F, Singhal B, Bacile EA, Saladino ML, Shaw C, Skromne E, Poehlau D, Vella N, Spelman T, Liew D, Kilpatrick TJ, Butzkueven H, Group MS (2013). Sex as a determinant of relapse incidence and progressive course of multiple sclerosis. Brain.

[5] Koch M, Kingwell E, Rieckmann P, Tremlett H, Neurologists UMC (2010). The natural history of secondary progressive multiple sclerosis. J Neurol Neurosurg Psychiatry.

[6] Confavreux C, Vukusic S, Adeleine P (2003). Early clinical predictors and progression of irreversible disability in multiple sclerosis: an amnesic process. Brain.

[7] Li R, Sun X, Shu Y, Mao Z, Xiao L, Qiu W, Lu Z, Hu X (2017). Sex differences in outcomes of disease-modifying treatments for multiple sclerosis: a systematic review. Mult Scler Relat Disord.

[8] Krysko KM, Bove R, Dobson R, Jokubaitis V, Hellwig K (2021). Treatment of women with multiple sclerosis planning pregnancy. Curr Treat Options Neurol.

[9] Krysko KM, Dobson R, Alroughani R, Amato MP, Bove R, Ciplea AI, Fragoso Y, Houtchens M, Jokubaitis VG, Magyari M, Abdelnasser A, Padma V, Thiel S, Tintore M, Vukusic S, Hellwig K (2023). Family planning considerations in people with multiple sclerosis. Lancet Neurol.

[10] Guger M, Enzinger C, Leutmezer F, Kraus J, Kalcher S, Kvas E, Berger T (2018). Real-life clinical use of natalizumab and fingolimod in Austria. Acta Neurol Scand.

[11] Thompson AJ, Banwell BL, Barkhof F, Carroll WM, Coetzee T, Comi G, Correale J, Fazekas F, Filippi M, Freedman MS, Fujihara K, Galetta SL, Hartung HP, Kappos L, Lublin FD, Marrie RA, Miller AE, Miller DH, Montalban X, Mowry EM, Sorensen PS, Tintore M, Traboulsee AL, Trojano M, Uitdehaag BMJ, Vukusic S, Waubant E, Weinshenker BG, Reingold SC, Cohen JA (2018). Diagnosis of multiple sclerosis: 2017 revisions of the McDonald criteria. Lancet Neurol.

[12] Polman CH, Reingold SC, Banwell B, Clanet M, Cohen JA, Filippi M, Fujihara K, Havrdova E, Hutchinson M, Kappos L, Lublin FD, Montalban X, O'Connor P, Sandberg-Wollheim M, Thompson AJ, Waubant E, Weinshenker B, Wolinsky JS (2011). Diagnostic criteria for multiple sclerosis: 2010 revisions to the McDonald criteria. Ann Neurol.

[13] Polman CH, Reingold SC, Edan G, Filippi M, Hartung HP, Kappos L, Lublin FD, Metz LM, McFarland HF, O'Connor PW, Sandberg-Wollheim M, Thompson AJ, Weinshenker BG, Wolinsky JS (2005). Diagnostic criteria for multiple sclerosis: 2005 revisions to the "McDonald Criteria". Ann Neurol.

[14] Wiendl H, Gold R, Berger T, Derfuss T, Linker R, Mäurer M, Aktas O, Baum K, Berghoff M, Bittner S, Chan A, Czaplinski A, Deisenhammer F, Di Pauli F, Du Pasquier R, Enzinger C, Fertl E, Gass A, Gehring K, Gobbi C, Goebels N, Guger M, Haghikia A, Hartung HP, Heidenreich F, Hoffmann O, Kallmann B, Kleinschnitz C, Klotz L, Leussink VI, Leutmezer F, Limmroth V, Lünemann JD, Lutterotti A, Meuth SG, Meyding-Lamadé U, Platten M, Rieckmann P, Schmidt S, Tumani H, Weber F, Weber MS, Zettl UK, Ziemssen T, Zipp F, (MSTCG) MSTCG (2021). Multiple sclerosis therapy consensus group (MSTCG): position statement on disease-modifying therapies for multiple sclerosis (white paper). Ther Adv Neurol Disord.

[15] Giovannoni G, Lang S, Wolff R, Duffy S, Hyde R, Kinter E, Wakeford C, Sormani MP, Kleijnen J (2020). A systematic review and mixed treatment comparison of pharmaceutical interventions for multiple sclerosis. Neurol Ther.

[16] Montalban X, Gold R, Thompson AJ, Otero-Romero S, Amato MP, Chandraratna D, Clanet M, Comi G, Derfuss T, Fazekas F, Hartung HP, Havrdova E, Hemmer B, Kappos L, Liblau R, Lubetzki C, Marcus E, Miller DH, Olsson T, Pilling S, Selmaj K, Siva A, Sorensen PS, Sormani MP, Thalheim C, Wiendl H, Zipp F (2018). ECTRIMS/EAN Guideline on the pharmacological treatment of people with multiple sclerosis. Mult Scler.

[17] Rae-Grant A, Day GS, Marrie RA, Rabinstein A, Cree BAC, Gronseth GS, Haboubi M, Halper J, Hosey JP, Jones DE, Lisak R, Pelletier D, Potrebic S, Sitcov C, Sommers R, Stachowiak J, Getchius TSD, Merillat SA, Pringsheim T (2018). Practice guideline recommendations summary: Disease-modifying therapies for adults with multiple sclerosis: report of the guideline development, dissemination, and implementation subcommittee of the american academy of neurology. Neurology.

[18] Cree BA, Gourraud PA, Oksenberg JR, Bevan C, Crabtree-Hartman E, Gelfand JM, Goodin DS, Graves J, Green AJ, Mowry E, Okuda DT, Pelletier D, von Büdingen HC, Zamvil SS, Agrawal A, Caillier S, Ciocca C, Gomez R, Kanner R, Lincoln R, Lizee A, Qualley P, Santaniello A, Suleiman L, Bucci M, Panara V, Papinutto N, Stern WA, Zhu AH, Cutter GR, Baranzini S, Henry RG, Hauser SL, University of California SnFM-ET (2016). Long-term evolution of multiple sclerosis disability in the treatment era. Ann Neurol.

[19] Wattjes MP, Ciccarelli O, Reich DS, Banwell B, de Stefano N, Enzinger C, Fazekas F, Filippi M, Frederiksen J, Gasperini C, Hacohen Y, Kappos L, Li DKB, Mankad K, Montalban X, Newsome SD, Oh J, Palace J, Rocca MA, Sastre-Garriga J, Tintoré M, Traboulsee A, Vrenken H, Yousry T, Barkhof F, Rovira À, Group MRIiMSs, Centres CoMS, Group NAIiMSCMgw (2021). 2021 MAGNIMS-CMSC-NAIMS consensus recommendations on the use of MRI in patients with multiple sclerosis. Lancet Neurol.

[20] Burnham KP, Anderson DR (2010). Model selection and multimodel inference: a practical information-theoretic approach.

[21] Belsley DA, Kuh E, Welsch RE (1980). Regression diagnostics: identifying influential data and sources of collinearity.

[22] O'Quigley J, Xu R, Stare J (2005). Explained randomness in proportional hazards models. Stat Med.

[23] Holm S (1979). A simple sequentially rejective multiple test procedure. Scand J Stat.

[24] R Core Team Vienna A (2021) A language and environment for statistical computing. In: R Foundation for Statistical Computing

[25] Qiu W, Chavarro J, Lazarus R, Rosner B, Ma J (2021) Power and sample size calculation for survival analysis of epidemiological studies. Rpackage version 0.1.3

[26] Sorensen PS, Kopp TI, Joensen H, Olsson A, Sellebjerg F, Magyari M (2021). Age and sex as determinants of treatment decisions in patients with relapsing-remitting MS. Mult Scler Relat Disord.

[27] Buron MD, Chalmer TA, Sellebjerg F, Barzinji I, Danny B, Christensen JR, Christensen MK, Hansen V, Illes Z, Jensen HB, Kant M, Papp V, Petersen T, Prakash S, Rasmussen PV, Schäfer J, Theódórsdóttir Á, Weglewski A, Sorensen PS, Magyari M (2020). Initial high-efficacy disease-modifying therapy in multiple sclerosis: a nationwide cohort study. Neurology.

[28] Moccia M, Palladino R, Carotenuto A, Russo CV, Triassi M, Lanzillo R, Brescia Morra V (2016). Predictors of long-term interferon discontinuation in newly diagnosed relapsing multiple sclerosis. Mult Scler Relat Disord.

[29] Meyniel C, Spelman T, Jokubaitis VG, Trojano M, Izquierdo G, Grand'Maison F, Oreja-Guevara C, Boz C, Lugaresi A, Girard M, Grammond P, Iuliano G, Fiol M, Cabrera-Gomez JA, Fernandez-Bolanos R, Giuliani G, Lechner-Scott J, Cristiano E, Herbert J, Petkovska-Boskova T, Bergamaschi R, van Pesch V, Moore F, Vella N, Slee M, Santiago V, Barnett M, Havrdova E, Young C, Sirbu CA, Tanner M, Rutherford M, Butzkueven H, Group MS (2012). Country, sex, EDSS change and therapy choice independently predict treatment discontinuation in multiple sclerosis and clinically isolated syndrome. PLoS One.

[30] Lu E, Wang BW, Guimond C, Synnes A, Sadovnick D, Tremlett H (2012). Disease-modifying drugs for multiple sclerosis in pregnancy: a systematic review. Neurology.

[31] Hellwig K, Tokic M, Thiel S, Esters N, Spicher C, Timmesfeld N, Ciplea AI, Gold R, Langer-Gould A (2022). Multiple sclerosis disease activity and disability following discontinuation of natalizumab for pregnancy. JAMA Netw Open.

[32] Hellwig K, Tokic M, Thiel S, Hemat S, Timmesfeld N, Ciplea AI, Gold R, Langer-Gould AM (2023). Multiple sclerosis disease activity and disability following cessation of fingolimod for pregnancy. Neurol Neuroimmunol Neuroinflamm.

[33] Dobson R, Hellwig K (2021). Use of disease-modifying drugs during pregnancy and breastfeeding. Curr Opin Neurol.

[34] Juto A, Fink K, Al Nimer F, Piehl F (2020). Interrupting rituximab treatment in relapsing-remitting multiple sclerosis; no evidence of rebound disease activity. Mult Scler Relat Disord.

[35] Bsteh G, Hegen H, Riedl K, Altmann P, Di Pauli F, Ehling R, Zulehner G, Rommer P, Leutmezer F, Deisenhammer F, Berger T (2021). Estimating risk of multiple sclerosis disease reactivation in pregnancy and postpartum: the VIPRiMS score. Front Neurol.

[36] Houtchens MK, Bove R (2018). A case for gender-based approach to multiple sclerosis therapeutics. Front Neuroendocrinol.

[37] Alonso-Moreno M, Ladrón-Guevara M, Ciudad-Gutiérrez P (2021) Systematic review of gender bias in clinical trials of monoclonal antibodies for the treatment of multiple sclerosis. Neurologia (Engl Ed) 10.1016/j.nrleng.2021.01.008 37996214

