# Supplementary material for: Sex impacts treatment decisions in multiple sclerosis
Source: J Neurol. 2024 Mar 5;271(6):3256–67. doi: 10.1007/s00415-024-12270-y (PMC11136719; doi:10.1007/s00415-024-12270-y)
Supplement: Supplementary file 1 — Supplementary file1 (PDF 2231 kb) [file 415_2024_12270_MOESM1_ESM.pdf]

Supplemental

Sex impacts Treatment Decisions in Multiple Sclerosis

Harald Hegen, Klaus Berek, Florian Deisenhammer, Thomas Berger, Christian Enzinger, Michael Guger, Jörg Kraus, Janette Walde, Franziska Di Pauli

**Figure e-1**	Time to start of DMT in female and male pwMS depending on different disease activity measures. ***Page 2***
**Table e-1:**	Demographics and clinical characteristics in pwMS depending on sex (*total cohort*). ***Page 3***
**Table e-2:**	Logistic regression analysis identifying predictors of highly effective DMT. ***Page 4***
**Table e-3:**	Gamma regression analysis identifying predictors of time to DMT. ***Page 5***
**Table e-4:**	Demographics and clinical characteristics in pwMS depending on treatment escalation (Question 1). ***Page 6***
**Table e-5:**	Demographics and clinical characteristics depending on sex in pwMS escalating or continuing DMT (*Question 1*). ***Page 7***
**Table e-6:**	Demographics and clinical characteristics in pwMS discontinuing moderate-efficacy DMT (*Question 2*). ***Page 8***
**Table e-7:**	Demographics and clinical characteristics depending on sex in pwMS stopping or continuing moderate-efficacy DMT (*Question 2*). ***Page 9***
**Table e-8:**	Reasons for discontinuation of moderate-efficacy DMT depending on sex (*Question 2*). ***Page 10***
**Table e-9:**	Demographics and clinical characteristics in pwMS depending in treatment de-escalation (*Question 3*). ***Page 11***
**Table e-10:**	Demographics and clinical characteristics depending on sex in pwMS de-escalating or continuing DMT (*Question 3*). ***Page 12***
**Table e-11:**	Demographics and clinical characteristics in pwMS discontinuing high-efficacy DMT (*Question 4*). ***Page 13***
**Table e-12:**	Demographics and clinical characteristics depending on sex in pwMS stopping or continuing high-efficacy DMT (*Question 4*). ***Page 14***
**Table e-13:**	Reasons for discontinuation of high-efficacy DMT depending on sex (Q*uestion 4*). ***Page 15***
**Table e-14:**	Cox regression analysis in a subgroup of pwMS excluding females with family planning for identifying predictors of early moderate-efficacy DMT discontinuation. ***Page 16***
**Table e-15:**	Cox regression analysis in the subgroup of pwMS excluding females with family planning for identifying predictors of early high-efficacy DMT discontinuation. ***Page 17***
**Table e-16:**	Sensitivity analysis in pwMS escalating or continuing DMT (*Question 1*) according to start of DMT. ***Page 18***
**Table e-17:**	Sensitivity analysis in pwMS discontinuing moderate-efficacy DMT (*Question 2*) according to start of DMT. ***Page 19***
**Table e-18:**	Sensitivity analysis in pwMS de-escalating or continuing high-efficacy DMT (*Question 3*) according to start of DMT. ***Page 20***
**Table e-19:**	Sensitivity analysis in pwMS discontinuing high-efficacy DMT (*Question 4*) according to start of DMT. ***Page 21***
**Table e-20**:	A posteriori power analyses for the multivariable Cox regression identifying predictors of DMT escalation (*Question 1*). ***Page 22***
**Table e-21**:	A posteriori power analyses for the multivariable Cox regression identifying predictors of moderate-efficacy DMT discontinuation (*Question 2*). ***Page 23***
**Table e-22**:	A posteriori power analyses for the multivariable Cox regression identifying predictors of DMT de-escalation (*Question 3*). ***Page 24***
**Table e-23**:	A posteriori power analyses for the multivariable Cox regression identifying predictors of high-efficacy DMT discontinuation (*Question 4*). ***Page 25***

Figure e-1 Time to start of DMT in female and male pwMS depending on different disease activity measures.

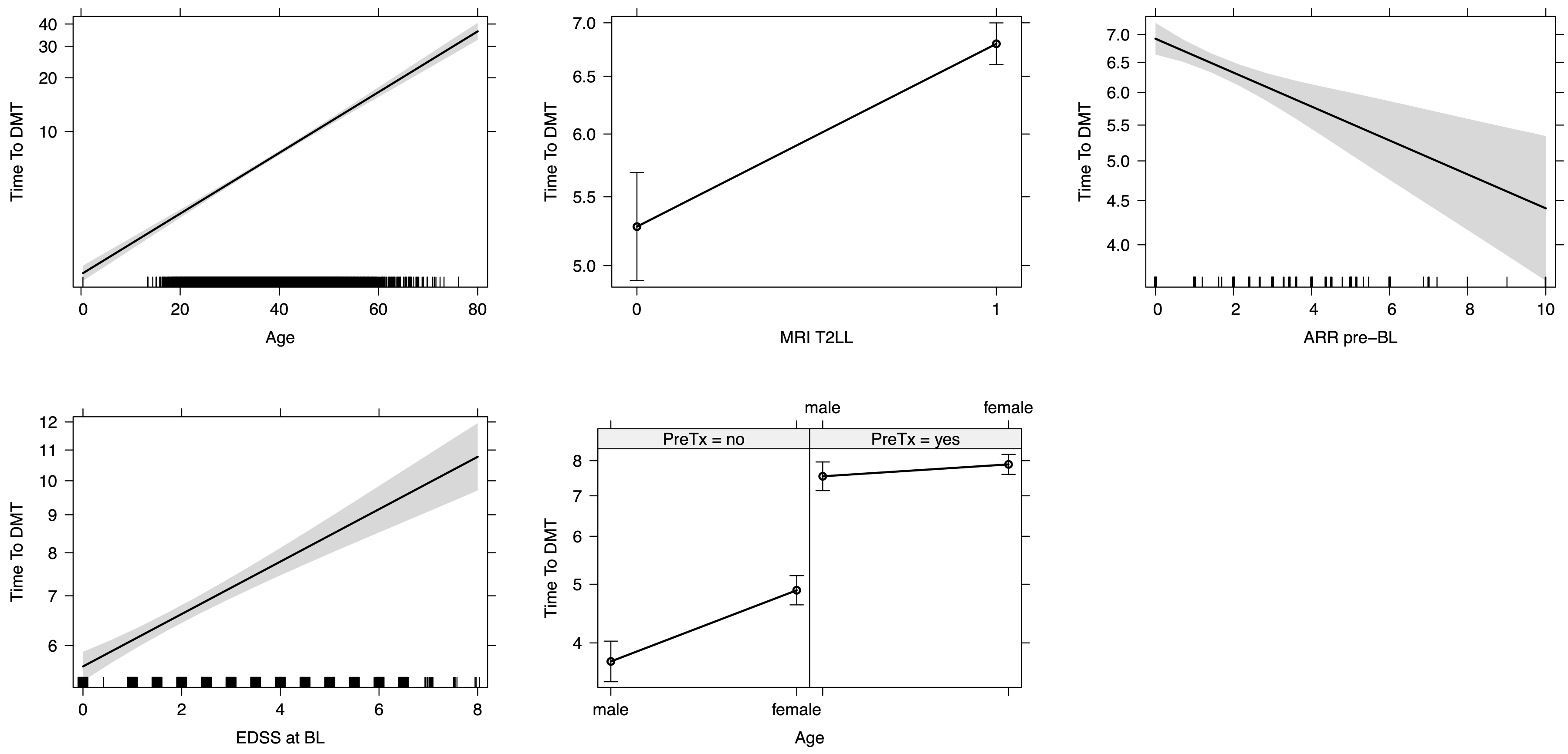

Legend:

Pre-treatment included interferon-beta and/ or glatiramer acetate.

Abbreviations: ARR, annualized relapse rate; BL, baseline; DMT, disease-modifying treatment; moderate-efficacy DMT, mDMT; MRI, magnetic resonance imaging; preTX, pre-treatment; T2LL, T2 lesions load

Table e-1: Demographics and clinical characteristics in pwMS depending on sex (*total cohort*)

	**All**	**Female**	**Male**
**Number**	4224	2905	1319
**Age** (years)	36.5 (28.9 – 45.0)	36.3 (28.7 – 44.7)	37.1 (29.5 – 45.4)
**Disease duration** (years)	5.9 (2.2 – 11.7)	6.0 (2.3 – 11.9)	5.6 (1.9 – 10.9)
**Year of disease onset**	2008 (2002-2013)	2007 (2001-2013)	2008 (2003-2013)
**Pre-ARR**[†,‖]	1 (1 – 2)	1 (1 – 2)	1 (1 – 2)
**EDSS**[§]	2 (1 – 3)	2 (1 – 3)	2 (1 – 3.5)
**MRI T2LL**[§] (>9), n (%)[‖]	3738 (88.5)	2567 (88.4)	1171 (88.8)
**Pre-treatment**[¶], n (%)	2989 (70.8)	2088 (71.9)	901 (68.3)
**DMT,** n (%)			
Dimethyl fumarate	1045 (24.7)	716 (24.6)	329 (24.9)
Teriflunomide	387 (9.2)	246 (8.5)	141 (10.7)
Fingolimod	1145 (27.1)	792 (27.3)	353 (26.8)
Natalizumab	1490 (35.3)	1058 (36.4)	432 (32.8)
Ocrelizumab	87 (2.1)	44 (1.5)	43 (3.3)
Cladribine	38 (0.9)	29 (1.0)	9 (0.7)
Alemtuzumab	32 (0.8)	20 (0.7)	12 (0.9)

Legend:
Data are shown as median (IQR) unless otherwise specified. Disease duration was the time between symptom onset and inclusion into registry, i.e. DMT start (baseline).
[§] These variables were assessed at baseline. [†] ARR was determined in the 12 months prior to baseline. [¶] Pre-treatment included interferon-beta and/ or glatiramer acetate.
[‖] Missing values: Pre-ARR, n=3; MRT T2LL, n=33.

Abbreviations: ARR, annualized relapse rate; DMT, disease-modifying treatment, MRI, magnetic resonance imaging; pwMS, people with multiple sclerosis; T2LL, T2 lesions load

Table e-2: Logistic regression analysis identifying predictors of highly effective DMT.

	**Coefficient**	**SE**	**P value**	**95% CI**		**OR**
**Constant**	-1.890	0.212	**<0.001**	-2.309	-1.476	0.151
**Sex** (female, ref: male)	-0.002	0.086	0.979	-0.172	0.166	0.998
**Age** [years]	-0.043	0.005	**<0.001**	-0.051	-0.034	0.958
**Disease duration** [years]	0.006	0.007	0.381	-0.007	0.019	1.006
**Pre-ARR**†	1.000	0.053	**<0.001**	0.898	1.106	2.719
**EDSS**§	0.511	0.033	**<0.001**	0.447	0.576	1.666
**MRI T2LL**§ (>9, ref: ≤9)	0.740	0.125	**<0.001**	0.496	0.985	2.095
**Pre-treatment**¶ (yes, ref: no)	1.571	0.088	**<0.001**	1.400	1.744	4.813

Cox Snell: 0.427

Legend:
§ These variables were assessed at baseline. † ARR was determined in the 12 months prior to baseline. ¶ Pre-treatment included interferon-beta and/ or glatiramer acetate.
[ ] shows units and () indicates reference categories.
Bold p-values hold with Bonferroni-Holm correction.

Abbreviations: ARR, annualized relapse rate; CI, confidence interval; DMT, disease-modifying treatment; EDSS, Expanded Disability Status Scale; MRI, magnetic resonance imaging; OR, Odds ratio; ref, reference; SE, standard error; T2LL, T2 lesions load

Table e-3: Gamma regression analysis identifying predictors of time to DMT.

	Estimate	SE	P value	95% CI		Exp(Estimate)
**Constant**	-0.456	0.069	**<0.001**	-0.596	-0.316	0.634
**Sex** (female, ref: male)	0.271	0.048	**<0.001**	0.176	0.365	1.311
**Age** [years]	0.039	0.001	**<0.001**	0.037	0.042	1.040
**Pre-ARR**†	-0.045	0.011	**<0.001**	-0.067	-0.023	0.956
**EDSS**§	0.081	0.009	**<0.001**	0.064	0.098	1.085
**MRI T2LL**§ (>9, ref: ≤9)	0.253	0.040	**<0.001**	0.174	0.332	1.288
**Pre-treatment**¶ (yes, ref: no)	0.704	0.048	**<0.001**	0.610	0.797	2.022
**Sex : Pre-treatment**	-0.225	0.058	**<0.001**	-0.339	-0.112	0.798

Cox Snell: 0.312

Legend:

§ These variables were assessed at baseline. † ARR was determined in the 12 months prior to baseline. ¶ Pre-treatment included interferon-beta and/ or glatiramer acetate.
[ ] shows units and () indicates reference categories.
Bold p-values hold with Bonferroni-Holm correction.

Abbreviations: ARR, annualized relapse rate; CI, confidence interval; DMT, disease-modifying treatment; EDSS, Expanded Disability Status Scale; MRI, magnetic resonance imaging; ref, reference; SE, standard error; T2LL, T2 lesions load
Note: Exp(estimate) gives the factor change in the expected value of y when x changes by 1 unit

Table e-4: Demographics and clinical characteristics in pwMS depending on treatment escalation (*Question 1*).

**DMT escalation**			
	**All**	**Escalation**	**No Escalation**
**Number**	1211	149	1062
**Sex** (female), n (%)	799 (66.0)	96 (64.4)	703 (66.2)
**Age** (years)	37.6 (29.9 – 46.1)	35.6 (27.7 – 42.8)	38.2 (30.1 – 46.6)
**Disease duration** (years)	4.1 (1.1 – 10.5)	3.4 (1.0 – 9.8)	4.3 (1.1 – 10.6)
**Year of disease onset**	2013 (2006-2016)	2013 (2006-2015)	2013 (2006-2017)
**Pre-ARR**[†]	1 (0 – 1)	1 (1 – 2)	1 (0 – 1)
**EDSS**[§]	1.5 (1 – 2)	1.5 (1 – 2)	1.5 (1 – 2)
**MRI T2LL**[§] (>9), n (%)	998 (82.4)	125 (83.9)	873 (82.2)
**Pre-treatment**[¶], n (%)	577 (47.6)	75 (50.3)	502 (47.3)
**DMT,** n (%)			
Dimethyl fumarate	881 (72.7)	99 (66.4)	782 (73.6)
Teriflunomide	330 (27.3)	50 (33.6)	280 (26.4)
**Time to DMT escalation (or observation period in pwMS without escalation)**	1.99 (0.96 – 3.41)	1.39 (0.71 – 2.45)	2.10 (1.01 – 3.60)
**ARR on DMT**[‡]	0.21 (0.51)	0.76 (0.90)	0.13 (0.38)
**EDSS Progression,** n (%)	225 (18.6)	61 (40.9)	164 (15.4)

Legend:
Data are shown as median (IQR) unless otherwise specified. Disease duration was the time between symptom onset and inclusion into registry, i.e. DMT start (baseline). ARR was calculated as the number of relapses divided by the observation period in years.

[§] These variables were assessed at baseline. [†] ARR was determined in the 12 months prior to baseline. [¶] Pre-treatment included interferon-beta and/ or glatiramer acetate., [‡] Mean (SD) is shown.

Abbreviations: ARR, annualized relapse rate; DMT, disease-modifying treatment; EDSS, Expanded Disability Status Scale; MRI, magnetic resonance imaging; pwMS, people with multiple sclerosis; T2LL, T2 lesions load

Table e-5: Demographics and clinical characteristics depending on sex in pwMS escalating or continuing DMT (*Question 1*).

	**All**	**Female**	**Male**
**Number**	1211	799	412
**Age** (years)	37.6 (29.9 – 46.1)	37.9 (30.2 – 46.0)	37.2 (29.6 – 46.3)
**Disease duration** (years)	4.1 (1.1 – 10.5)	4.6 (1.2 – 11.3)	3.4 (1.0 – 9.1)
**Year of disease onset**	2013 (2006-2016)	2012 (2006-2016)	2013 (2008-2017)
**Pre-ARR**[†]	1 (0 – 1)	1 (0 – 1)	1 (1 – 1)
**EDSS**[§]	1.5 (1 – 2)	1.5 (1 – 2)	1.5 (1 – 2)
**MRI T2LL**[§] (>9), n (%)	998 (82.4)	656 (82.1)	342 (83.0)
**Pre-treatment**[¶], n (%)	577 (47.6)	393 (49.2)	184 (44.7)
**DMT,** n (%)			
Dimethyl fumarate	881 (72.7)	591 (73.9)	290 (70.4)
Teriflunomide	330 (27.3)	208 (26.0)	122 (29.6)
**Time to DMT escalation (or observation period in pwMS without escalation)**	1.99 (0.96 – 3.41)	1.94 (0.96-3.41)	2.12 (0.98-3.41)
**ARR on DMT**[‡]	0.21 (0.51)	0.21 (0.52)	0.22 (0.50)
**EDSS Progression,** n (%)	225 (18.6)	150 (18.8)	75 (18.2)

Legend:
Data are shown as median (IQR) unless otherwise specified. Disease duration was the time between symptom onset and inclusion into registry, i.e. DMT start (baseline).
[§] These variables were assessed at baseline. [†] ARR was determined in the 12 months prior to baseline. [¶] Pre-treatment included interferon-beta and/ or glatiramer acetate.
[‡] Mean (SD) is shown.

Abbreviations: ARR, annualized relapse rate; DMT, disease-modifying treatment, EDSS, Expanded Disability Status Scale, MRI, magnetic resonance imaging; pwMS, people with multiple sclerosis; T2LL, T2 lesions load

Table e-6: Demographics and clinical characteristics in pwMS discontinuing moderate-efficacy DMT (*Question 2*).

mDMT discontinuation			
	**All**	**Discontinuation**	**No Discontinuation**
**Number**	862	73	789
**Sex** (female), n (%)	578 (67.1)	59 (80.8)	519 (65.8)
**Age** (years)	38.6 (30.4 – 46.8)	35.9 (26.7 – 49.3)	38.8 (31.0 – 46.5)
**Disease duration** (years)	4.8 (1.3 – 10.9)	5.2 (1.7 – 9.6)	4.8 (1.3 – 10.9)
**Year of disease onset**	2012 (2006-2015)	2011 (2007-2014)	2012 (2006-2015)
**Pre-ARR**†	1 (0 – 1)	1 (0 – 1)	1 (0 – 1)
**EDSS**§	1.5 (1.0 – 2.0)	2.0 (1.0 – 2.5)	1.5 (1.0 – 2.0)
**MRI T2LL**§ (>9), n (%)	719 (83.4)	61 (83.6)	658 (83.4)
**Pre-treatment**¶, n (%)	434 (50.3)	43 (58.9)	391 (49.6)
**DMT**, n (%)			
Dimethyl fumarate	640 (74.2)	58 (79.5)	582 (73.8)
Teriflunomide	222 (25.8)	15 (20.5)	207 (26.2)
**Time to DMT discontinuation (or observation period in pwMS without discontinuation)**	2.7 (1.8 – 4.0)	2.4 (1.9 – 3.4)	2.7 (1.8 – 4.1)
**ARR on DMT**‡	0.11 (0.26)	0.06 (0.19)	0.12 (0.27)
**EDSS progression**, n (%)	65 (7.5)	12 (16.4)	53 (6.7)

Legend:
Data are shown as median (IQR) unless otherwise specified. Disease duration was the time between symptom onset and inclusion into registry, i.e. DMT start (baseline). ARR was calculated as the number of relapses divided by the observation period in years.

§ These variables were assessed at baseline. † ARR was determined in the 12 months prior to baseline. ¶ Pre-treatment included interferon-beta and/ or glatiramer acetate., ‡ Mean (SD) is shown.

Abbreviations: ARR, annualized relapse rate; DMT, disease-modifying treatment; EDSS, Expanded Disability Status Scale; mDMT, moderate-efficacy DMT; MRI, magnetic resonance imaging; pwMS, people with multiple sclerosis; T2LL, T2 lesions load

Table e-7: Demographics and clinical characteristics depending on sex in pwMS stopping or continuing moderate-efficacy DMT (*Question 2*).

	**All**	**Female**	**Male**
**Number**	862	578	284
**Age** (years)	38.6 (30.4 – 46.8)	38.5 (30.2 – 46.2)	38.8 (30.9 – 47.9)
**Disease duration** (years)	4.8 (1.3 – 10.9)	5.3 (1.5 – 11.3)	4.0 (1.1 – 9.8)
**Year of disease onset**	2012 (2006-2015)	2011 (2005-2015)	2012 (2007-2016)
**Pre-ARR**†	1 (0 – 1)	1 (0 – 1)	1 (0 – 1)
**EDSS**§	1.5 (1.0 – 2.0)	1.5 (1.0 – 2.0)	1.5 (1.0 – 2.0)
**MRI T2LL**§ (>9), n (%)	719 (83.4)	484 (83.7)	235 (82.7)
**Pre-treatment**¶, n (%)	434 (50.3)	299 (51.7)	135 (47.5)
**DMT,** n (%)			
Dimethyl fumarate	640 (74.2)	433 (74.9)	207 (72.9)
Teriflunomide	222 (25.8)	145 (25.1)	77 (27.1)
**Time to DMT discontinuation (or observation period in pwMS without discontinuation)**	2.7 (1.8 – 4.0)	2.6 (1.7-4.1)	2.8 (2.0-4.0)
**ARR on DMT**‡	0.11 (0.26)	0.12 (0.27)	0.10 (0.24)
**EDSS progression**, n (%)	65 (7.5)	44 (7.6)	21 (7.4)

Legend:
Data are shown as median (IQR) unless otherwise specified. Disease duration was the time between symptom onset and inclusion into registry, i.e. DMT start (baseline). ARR was calculated as the number of relapses divided by the observation period in years.

§ These variables were assessed at baseline. † ARR was determined in the 12 months prior to baseline. ¶ Pre-treatment included interferon-beta and/ or glatiramer acetate. ‡ Mean (SD) is shown.

Abbreviations: ARR, annualized relapse rate; DMT, disease-modifying treatment; EDSS, Expanded Disability Status Scale; mDMT, moderate-efficacy DMT; MRI, magnetic resonance imaging; pwMS, people with multiple sclerosis; T2LL, T2 lesions load

Table e-8: Reasons for discontinuation of moderate-efficacy DMT depending on sex (*Question 2*).

	**All**	**Female**	**Male**
Family planning	24 (33)	24 (100)	0 (0)
Patient's request	19 (26)	15 (79)	4 (21)
Disease stability	8 (11)	6 (75)	2 (25)
Disease progression	5 (7)	1 (20)	4 (80)
Adverse events	17 (23)	15 (88)	2 (12)

Legend:
Data are shown as number (percentage).

Abbreviations: DMT, disease-modifying treatment

Table e-9: Demographics and clinical characteristics in pwMS depending in treatment de-escalation (*Question 3*).

**DMT de-escalation**			
	**All**	**De-escalation**	**No de-escalation**
**Number**	1836	78	1758
**Sex** (female), n (%)	1268 (69.1)	48 (61.5)	1220 (69.4)
**Age** (years)	35.8 (28.3 – 43.4)	34.1 (29.5 – 44.7)	35.8 (28.2 – 43.4)
**Disease duration** (years)	6.8 (3.2 – 12.3)	7.5 (3.9 – 13.0)	6.8 (3.2 – 12.2)
**Year of disease onset**	2006 (2000-2010)	2003 (2000-2009)	2006 (2000-2010)
**Pre-ARR**†	2 (1 – 2)	2 (1 – 2)	2 (1 – 2)
**EDSS**§	2.5 (1.5 – 3.5)	2 (1.5 – 3)	2.5 (1.5 – 3.5)
**MRI T2LL**§ (>9), n (%)	1696 (92.4)	73 (93.6)	1623 (92.3)
**Pre-treatment**¶, n (%)	1573 (85.7)	70 (89.7)	1503 (85.5)
**DMT,** n (%)			
Fingolimod	829 (45.2)	29 (37.2)	800 (45.5)
Natalizumab	1007 (54.8)	49 (62.8)	958 (54.5)
**Time to de-escalation (or observation period in pwMS without de-escalation)**	5.53 (3.24 – 7.89)	4.26 (2.21 – 6.84)	5.59 (3.30 – 7.92)
**ARR on DMT**‡	0.16 (0.29)	0.16 (0.26)	0.16 (0.29)
**EDSS progression**, n (%)	629 (34.3)	20 (25.6)	609 (34.6)

Legend:
Data are shown as median (IQR) unless otherwise specified. Disease duration was the time between symptom onset and inclusion into registry, i.e. DMT start (baseline). ARR was calculated as the number of relapses divided by the observation period in years.

§ These variables were assessed at baseline. † ARR was determined in the 12 months prior to baseline. ¶ Pre-treatment included interferon-beta and/ or glatiramer acetate., ‡ Mean (SD) is shown.

Abbreviations: ARR, annualized relapse rate; DMT, disease-modifying treatment; EDSS, Expanded Disability Status Scale; MRI, magnetic resonance imaging; pwMS, people with multiple sclerosis; T2LL, T2 lesions load

Table e-10: Demographics and clinical characteristics depending on sex in pwMS de-escalating or continuing DMT (*Question 3*).

	**All**	**Female**	**Male**
**Number**	1836	1268	568
**Age** (years)	35.8 (28.3 – 43.4)	36.3 (28.4 – 43.7)	34.4 (28.0 – 42.9)
**Disease duration** (years)	6.8 (3.2 – 12.3)	7.2 (3.2 – 12.9)	6.1 (3.0 – 11.0)
**Year of disease onset**	2006 (2000-2010)	2005 (1999-2010)	2006 (2002-2010)
**Pre-ARR**†	2 (1 – 2)	2 (1 – 2)	2 (1 – 2)
**EDSS**§	2.5 (1.5 – 3.5)	2 (1.5 – 3.5)	2.5 (1.5 – 3.5)
**MRI T2LL**§ (>9), n (%)	1696 (92.4)	1172 (92.4)	524 (92.3)
**Pre-treatment**¶, n (%)	1573 (85.7)	1087 (85.7)	486 (85.6)
**DMT,** n (%)			
Fingolimod	829 (45.2)	564 (44.5)	265 (46.7)
Natalizumab	1007 (54.8)	704 (55.5)	303 (53.3)
**Time to de-escalation (or observation period in pwMS without de-escalation)**	5.53 (3.24 – 7.89)	5.58 (3.20-7.97)	5.51 (3.33-7.55)
**ARR on DMT**‡	0.16 (0.29)	0.17 (0.31)	0.14 (0.24)
**EDSS progression**, n (%)	629 (34.3)	429 (33.8)	200 (35.2)

Legend:
Data are shown as median (IQR) unless otherwise specified. Disease duration was the time between symptom onset and inclusion into registry, i.e. DMT start (baseline). ARR was calculated as the number of relapses divided by the observation period in years.

§ These variables were assessed at baseline. † ARR was determined in the 12 months prior to baseline. ¶ Pre-treatment included interferon-beta and/ or glatiramer acetate. ‡ Mean (SD) is shown.

Abbreviations: ARR, annualized relapse rate; DMT, disease-modifying treatment; EDSS, Expanded Disability Status Scale; MRI, magnetic resonance imaging; pwMS, people with multiple sclerosis; T2LL, T2 lesions load

Table e-11: Demographics and clinical characteristics in pwMS discontinuing high-efficacy DMT (*Question 4*).

**hDMT discontinuation**			
	**All**	**Discontinuation**	**No Discontinuation**
**Number**	1941	231	1710
**Sex** (female), n (%)	1370 (70.6)	186 (80.5)	1184 (69.2)
**Age** (years)	35.7 (28.2 – 43.6)	33.9 (28.0 – 43.8)	35.8 (28.2 – 43.5)
**Disease duration** (years)	6.8 (3.1 – 12.2)	6.6 (2.9 – 10.9)	6.8 (3.2 – 12.3)
**Year of disease onset**	2006 (2000-2010)	2005 (2000-2009)	2006 (2000-2010)
**Pre-ARR**†	2 (1 – 2)	2 (1 – 3)	2 (1 – 2)
**EDSS**§	2.5 (1.5 – 3.5)	2.5 (1.5 – 4.0)	2.3 (1.5 – 3.5)
**MRI T2LL**§ (>9), n (%)	1796 (92.5)	217 (93.9)	1579 (92.3)
**Pre-treatment**¶, n (%)	1660 (85.5)	197 (85.3)	1463 (85.6)
**DMT,** n (%)			
Natalizumab	1074 (55.3)	158 (68.4)	916 (53.6)
Fingolimod	867 (44.7)	73 (31.6)	794 (46.4)
**Time to DMT discontinuation (or observation period in pwMS without discontinuation)**	5.36 (3.08 – 7.72)	2.92 (1.95 – 5.09)	5.63 (3.32 – 7.92)
**ARR on DMT**‡	0.18 (0.34)	0.33 (0.59)	0.16 (0.29)
**EDSS progression**, n (%)	318 (16.4)	71 (30.7)	247 (14.4)

Legend:
Data are shown as median (IQR) unless otherwise specified. Disease duration was the time between symptom onset and inclusion into registry, i.e. DMT start (baseline). ARR was calculated as the number of relapses divided by the observation period in years.

§ These variables were assessed at baseline. † ARR was determined in the 12 months prior to baseline. ¶ Pre-treatment included interferon-beta and/ or glatiramer acetate., ‡ Mean (SD) is shown.

Abbreviations: ARR, annualized relapse rate; DMT, disease-modifying treatment, EDSS, Expanded Disability Status Scale; hDMT, high-efficacy DMT; MRI, magnetic resonance imaging; pwMS, people with multiple sclerosis; T2LL, T2 lesions load

Table e-12: Demographics and clinical characteristics depending on sex in pwMS stopping or continuing high-efficacy DMT (*Question 4*).

	**All**	**Female**	**Male**
**Number**	1941	1370	571
**Age** (years)	35.7 (28.2 – 43.6)	35.9 (28.3 – 43.5)	34.7 (28.0 – 43.8)
**Disease duration** (years)	6.8 (3.1 – 12.2)	7.1 (3.2 – 12.7)	6.2 (3.0 – 11.0)
**Year of disease onset**	2006 (2000-2010)	2005 (1999-2010)	2006 (2002-2010)
**Pre-ARR**†	2 (1 – 2)	2 (1 – 2)	2 (1 – 2)
**EDSS**§	2.5 (1.5 – 3.5)	2.0 (1.5 – 3.5)	2.5 (1.5 – 3.5)
**MRI T2LL**§ (>9), n (%)	1796 (92.5)	1268 (92.6)	528 (92.5)
**Pre-treatment**¶, n (%)	1660 (85.5)	1171 (85.5)	489 (85.6)
**DMT,** n (%)			
Natalizumab	1074 (55.3)	767 (56.0)	307 (53.8)
Fingolimod	867 (44.7)	603 (44.0)	264 (46.2)
**Time to DMT discontinuation (or observation period in pwMS without discontinuation)**	5.36 (3.08 – 7.72)	5.28 (2.97-7.74)	5.46 (3.34-7.52)
**ARR on DMT**‡	0.18 (0.34)	0.19 (0.37)	0.15 (0.26)
**EDSS progression**, n (%)	318 (16.4)	216 (15.8)	102 (17.9)

Legend:
Data are shown as median (IQR) unless otherwise specified. Disease duration was the time between symptom onset and inclusion into registry, i.e. DMT start (baseline). ARR was calculated as the number of relapses divided by the observation period in years.

§ These variables were assessed at baseline. † ARR was determined in the 12 months prior to baseline. ¶ Pre-treatment included interferon-beta and/ or glatiramer acetate. ‡ Mean (SD) is shown.

Abbreviations: ARR, annualized relapse rate; DMT, disease-modifying treatment; EDSS, Expanded Disability Status Scale; MRI, magnetic resonance imaging; pwMS, people with multiple sclerosis; T2LL, T2 lesions load

Table e-13: Reasons for discontinuation of high-efficacy DMT depending on sex (*Question 4*).

	**All**	**Female**	**Male**
Family planning	74 (32)	74 (100)	0 (0)
JCV positivity	32 (14)	25 (78)	7 (22)
Patient’s request	43 (19)	31 (72)	12 (28)
Disease stability	3 (1)	2 (67)	1 (33)
Disease progression	31 (13)	21 (68)	10 (32)
Adverse events	31 (13)	23 (74)	8 (26)
PML	7 (3)	6 (86)	1 (14)
Neutralizing antibodies	1 (0.4)	1 (100)	0 (0)
Other	9 (4)	3 (33)	6 (67)

Legend:
Data are shown as number (percentage).

Abbreviations: DMT, disease-modifying treatment; JCV, John Cunningham virus; PML, progressive multifocal leukoencephalopathy

Table e-14: Cox regression analysis in a subgroup of pwMS excluding females with family planning for identifying predictors of early moderate-efficacy DMT discontinuation.

	**Coefficient**	**SE**	**P value**
**Sex** (female, ref: male)	0.736	0.421	0.081
**Age** [years]	0.098	0.030	**<0.001**
**Disease duration** [years]	-0.039	0.022	0.075
**Pre-ARR**†	-0.176	0.213	0.410
**EDSS**§	0.059	0.111	0.595
**MRI T2LL**§ (>9, ref: ≤9)	-0.184	0.404	0.649
**Pre-treatment**¶ (yes, ref: no)	0.219	0.314	0.487
**DMT** (DMF, ref: TER)	0.734	0.350	0.036
**ARR on DMT**	-1.674	1.019	0.100
**EDSS progression** (yes, ref: no)	0.970	0.381	0.012
**Sex : Age**	-0.062	0.032	0.052

Legend:
§ These variables were assessed at baseline. † ARR was determined in the 12 months prior to baseline. ¶ Pre-treatment included interferon-beta and/ or glatiramer acetate.
[ ] shows units and () indicates reference categories.
Bold p-values hold with Bonferroni-Holm correction.

Abbreviations: ARR, annualized relapse rate; DMF, dimethyl fumarate; DMT, disease-modifying treatment; EDSS, Expanded Disability Status Scale; SE, standard error; T2LL, T2 lesions load; TER, teriflunomide

Table e-15: Cox regression analysis in the subgroup of pwMS excluding females with family planning for identifying predictors of early high-efficacy DMT discontinuation.

	**Coefficient**	**SE**	**P value**
**Sex** (female, ref: male)	0.040	0.181	0.825
**Age** [years]	0.028	0.009	0.003
**Disease duration** [years]	-0.008	0.013	0.539
**Pre-ARR**†	0.010	0.071	0.885
**EDSS**§	0.226	0.055	**<0.001**
**MRI T2LL**§ (>9, ref: ≤9)	0.220	0.391	0.573
**Pre-treatment**¶ (yes, ref: no)	-0.215	0.240	0.370
**DMT** (NTZ, ref: FTY)	0.204	0.200	0.309
**ARR on DMT**#	0.044	0.339	0.897
**EDSS progression** (yes, ref: no)	0.717	0.173	**<0.001**

Legend:

§ These variables were assessed at baseline. † ARR was determined in the 12 months prior to baseline. ¶ Pre-treatment included interferon-beta and/ or glatiramer acetate.
# Additionally, interaction between the variable “ARR on DMT” and different “reasons for DMT stop” (adverse events and patient request) was considered due to confounding.
[ ] shows units and () indicates reference categories.
Bold p-values hold with Bonferroni-Holm correction.

Abbreviations: ARR, annualized relapse rate; DMT, disease-modifying treatment; EDSS, Expanded Disability Status Scale; FTY, fingolimod; MRI, magnetic resonance imaging; NTZ, natalizumab; ref, reference; SE, standard error; T2LL, T2 lesions load

Table e-16: Sensitivity analysis in pwMS escalating or continuing DMT (*Question 1*) according to start of DMT.

	**Whole cohort**			**After 2015**
	**Coefficient**	**95%-CI**		**Coefficient**
**Age** [years]	-0.021	-0.041	-0.001	-0.024
**EDSS**§	0.144	0.003	0.286	0.139
**ARR on DMT**	2.117	1.779	2.455	2.013
**Sex : ARR on DMT**	-0.708	-1.073	-0.344	-0.636
**EDSS progression** (yes, ref: no)	0.417	0.069	0.765	0.515
**MRI activity during DMT** (yes, ref: no)	3.166	2.546	3.786	3.286

Legend:
For this sensitivity analysis, the cohort of patients were used when inclusion into the AMSTR was after January, 1st 2015. For patients before 2015, separate analysis is not shown due to sample size limits.
Absolute numbers of events (DMT escalation from TER or DMF) was low before 2015, as both DMT have been approved not before 2013 and 2014, respectively. Note that the percentage of events (DMT escalation) was similar before/ after January, 1st 2015: 17/112 (15%) vs. 132/1099 (12%).
§ This variable was assessed at baseline.
[ ] shows units and () indicates reference categories.

Abbreviations: ARR, annualized relapse rate; CI, confidence interval; DMT, disease-modifying treatment; EDSS, Expanded Disability Status Scale; MRI, magnetic resonance imaging.

Table e-17: Sensitivity analysis in pwMS discontinuing moderate-efficacy DMT (*Question 2*) according to start of DMT.

	**Whole cohort**			**After 2015**
	**Coefficient**	**95%-CI**		**Coefficient**
**Sex** (female, ref: male)	1.214	0,421	2,007	1.303
**Age** [years]	0.087	0,032	0,142	0.057
**ARR on DMT**	-1.674	-3,287	-0,060	-1.312
**EDSS progression** (yes, ref: no)	0.773	0,131	1,416	0.833
**Sex : Age**	-0.112	-0,170	-0,055	-0.090

Legend:
For this sensitivity analysis, the cohort of patients were used when inclusion into the AMSTR was after January, 1st 2015. For patients before 2015, separate analysis is not shown due to sample size limits.
Absolute numbers of events (discontinuation of TER or DMF) was low before 2015, as both DMT have been approved not before 2013 and 2014, respectively. Note that the percentage of events (DMT discontinuation) was similar before/ after January, 1st 2015: 10/95 (11%) vs. 63/767 (8%).
[ ] shows units and () indicates reference categories.

Abbreviations: ARR, annualized relapse rate; CI, confidence interval; DMT, disease-modifying treatment; EDSS, Expanded Disability Status Scale.

Table e-18: Sensitivity analysis in pwMS de-escalating or continuing high-efficacy DMT (*Question 3*) according to start of DMT.

	**Whole cohort**			**Before 2015**
	**Coefficient**	**95%-CI**		**Coefficient**
**EDSS progression** (yes, ref: no)	-0.719	-1.240	-0.197	-0.653

Legend:
For this sensitivity analysis, the cohort of patients were used when inclusion into the AMSTR was before January, 1st 2015. For patients after 2015, separate analysis is not shown due to sample size limits.
Note that the percentage of events (DMT de-escalation) was similar before/ after January, 1st 2015: 67/1318 (5%) vs. 11/518 (2%)
() indicates reference categories.

Abbreviations: CI, confidence interval; EDSS, Expanded Disability Status Scale.

Table e-19: Sensitivity analysis in pwMS discontinuing high-efficacy DMT (*Question 4*) according to start of DMT.

	**Whole cohort**			**Before 2015**
	**Coefficient**	**95%-CI**		**Coefficient**
**Sex** (female, ref: male)	0.542	0.211	0.873	0.648
**EDSS§**	0.163	0.070	0.255	0.185
**ARR on DMT**	-0.958	-1.697	-0.217	-0.549
**EDSS progression** (yes, ref: no)	0.634	0.337	0.932	0.686

Legend:
For this sensitivity analysis, the cohort of patients were used when inclusion into the AMSTR was before January, 1st 2015. For patients after 2015, separate analysis is not shown due to sample size limits.
Note that the percentage of events (DMT escalation) was lower after January, 1st 2015: 37/542 (7%) vs. 194/1399 (14%).
() indicates reference categories.

Abbreviations: ARR, annualized relapse rate; CI, confidence interval; DMT, disease-modifying treatment; EDSS, Expanded Disability Status Scale

Table e-20: A posteriori power analyses for the multivariable Cox regression identifying predictors of DMT escalation (*Question 1*).

	Coefficient	HR	Sig.	A posteriori power	Sample size for a power of 0.8
**Sex** (female, ref: male)	0.446	1.562		0.8	1340
**Age** [years]	-0.021	0.979	*		
**Disease duration** [years]	-0.025	0.975		0.6	2162
**Pre-ARR**[†]	0.004	1.004		effect size of this co-variable not clinically relevant	
**EDSS**[§]	0.144	1.155	*		
**MRI T2LL**[§] (>9, ref: ≤9)	-0.021	0.979		effect size of this co-variable not clinically relevant	
**Pre-treatment**[¶] (yes, ref: no)	0.017	1.017		effect size of this co-variable not clinically relevant	
**DMT** (DMF, ref: TER)	-0.043	0.958		effect size of this co-variable not clinically relevant	
**ARR on DMT**	2.117	8.309	***		
**Sex : ARR on DMT**	-0.708	0.493	***		
**EDSS progression** (yes, ref: no)	0.417	1.517			
**MRI activity during DMT** (yes, ref: no)	3.166	23.709	***		

Legend:
[§] These variables were assessed at baseline. [†] ARR was determined in the 12 months prior to baseline. [¶] Pre-treatment included interferon-beta and/ or glatiramer acetate.

*, **, *** shows statistical significance at a p value <0.05, <0.01 and <0.001.
[ ] shows units and () indicates reference categories.
":" denotes interaction effects between variables.

Abbreviations: ARR, annualized relapse rate; DMF, dimethyl fumarate; DMT, disease-modifying treatment; EDSS, Expanded Disability Status Scale; HR, hazard ratio; MRI, magnetic resonance imaging; Sig., Significance; T2LL, T2 lesions load; TER, teriflunomide

Table e-21: A posteriori power analyses for the multivariable Cox regression identifying predictors of moderate-efficacy DMT discontinuation (*Question 2*).

	Coefficient	HR	Sig.	A posteriori power	Sample size for a power of 0.8
**Sex** (female, ref: male)	1.214	3.366	**		
**Age** [years]	0.087	1.091	**		
**Disease duration** [years]	-0.031	0.970		0.5	2032
**DMT** (DMF, ref: TER)	0.617	1.853		0.7	1115
**Pre-ARR**†	-0.027	0.973		effect size of this co-variable not clinically relevant	
**EDSS**§	0.132	1.141		0.4	3179
**MRI T2LL**§ (>9, ref: ≤9)	-0.032	0.968		effect size of this co-variable not clinically relevant	
**Pre-treatment**¶ (yes, ref: no)	0.337	1.400		0.4	2959
**ARR on DMT**	-1.674	0.188	*		
**EDSS progression** (yes, ref: no)	0.773	2.167	*		
**Sex : Age**	-0.112	0.894	***		

Legend:
§ These variables were assessed at baseline. † ARR was determined in the 12 months prior to baseline. ¶ Pre-treatment included interferon-beta and/ or glatiramer acetate.

*, **, *** shows statistical significance at a p value <0.05, <0.01 and <0.001.
[ ] shows units and () indicates reference categories.
":" denotes interaction effects between variables.

Abbreviations: ARR, annualized relapse rate; DMF, dimethyl fumarate; DMT, disease-modifying treatment; EDSS, Expanded Disability Status Scale; HR, hazard ratio; MRI, magnetic resonance imaging; pwMS, people with multiple sclerosis; Sig., Significance; T2LL, T2 lesions load; TER, teriflunomide

Table e-22: A posteriori power analyses for the multivariable Cox regression identifying predictors of DMT de-escalation (*Question 3*).

	Coefficient	HR	Sig.	A posteriori power	Sample size for a power of 0.8
**Sex** (female, ref: male)	-0.385	0.680		0.5	4648
**Age** [years]	0.014	1.014		effect size of this co-variable not clinically relevant	
**Disease duration** [years]	0.008	1.008		effect size of this co-variable not clinically relevant	
**Pre-ARR**†	-0.060	0.942		effect size of this co-variable not clinically relevant	
**EDSS**§	-0.069	0.933		effect size of this co-variable not clinically relevant	
**MRI T2LL**§ (>9, ref: ≤9)	0.076	1.079		effect size of this co-variable not clinically relevant	
**Pre-treatment**¶ (yes, ref: no)	0.119	1.126		effect size of this co-variable not clinically relevant	
**DMT** (NTZ, ref: FTY)	-0.058	0.943		effect size of this co-variable not clinically relevant	
**ARR on DMT**	0.292	1.339		effect size of this co-variable not clinically relevant	
**EDSS progression** (yes, ref: no)	-0.719	0.487	**		

Legend:
§ These variables were assessed at baseline. † ARR was determined in the 12 months prior to baseline. ¶ Pre-treatment included interferon-beta and/ or glatiramer acetate.

*, **, *** shows statistical significance at a p value <0.05, <0.01 and <0.001.
[ ] shows units and () indicates reference categories.

Abbreviations: ARR, annualized relapse rate; DMT, disease-modifying treatment; EDSS, Expanded Disability Status Scale; FTY, fingolimod; HR, hazard ratio; MRI, magnetic resonance imaging; NTZ, natalizumab; pwMS, people with multiple sclerosis; Sig., Significance; T2LL, T2 lesions load

Table e-23: A posteriori power analyses for the multivariable Cox regression identifying predictors of high-efficacy DMT discontinuation (*Question 4*).

	Coefficient	HR	Sig.	A posteriori power	Sample size for a power of 0.8
**Sex** (female, ref: male)	0.542	1.719	**		
**Age** [years]	0.001	1.001		effect size of this co-variable not clinically relevant	
**Disease duration** [years]	-0.021	0.979		0.6	3709
**Pre-ARR**†	-0.034	0.966		effect size of this co-variable not clinically relevant	
**EDSS**§	0.163	1.176	***		
**MRI T2LL**§ (>9, ref: ≤9)	0.030	1.031		effect size of this co-variable not clinically relevant	
**Pre-treatment**¶ (yes, ref: no)	-0.235	0.791		0.3	7807
**DMT** (NTZ, ref: FTY)	-0.009	0.991		effect size of this co-variable not clinically relevant	
**ARR on DMT**	-0.957	0.384	*		
**EDSS progression** (yes, ref: no)	0.634	1.886	***		

Legend:
§ These variables were assessed at baseline. † ARR was determined in the 12 months prior to baseline. ¶ Pre-treatment included interferon-beta and/ or glatiramer acetate.

*, **, *** shows statistical significance at a p value <0.05, <0.01 and <0.001.
[ ] shows units and () indicates reference categories.

Abbreviations: ARR, annualized relapse rate; DMT, disease-modifying treatment; EDSS, Expanded Disability Status Scale; FTY, fingolimod; HR, hazard ratio; MRI, magnetic resonance imaging; NTZ, natalizumab; pwMS, people with multiple sclerosis; Sig., Significance; T2LL, T2 lesions load
